# BDNF Activates Postsynaptic TrkB Receptors to Induce Endocannabinoid Release and Inhibit Presynaptic Calcium Influx at a Calyx-Type Synapse

**DOI:** 10.1523/JNEUROSCI.2838-19.2020

**Published:** 2020-10-14

**Authors:** Yichen Wu, Qingzhuo Liu, Bin Guo, Fangfei Ye, Jianlong Ge, Lei Xue

**Affiliations:** ^1^State Key Laboratory of Medical Neurobiology and MOE Frontiers Center for Brain Science, Department of Physiology and Biophysics, School of Life Sciences, Fudan University, Shanghai, P.R. China 200438; ^2^Department of Neurology, Children's Hospital of Fudan University, National Children's Medical Center, Shanghai, P.R. China 201102

**Keywords:** AC/PKA, BDNF, endocannabinoid, endocytosis, exocytosis, retrograde signaling

## Abstract

Brain-derived neurotropic factor (BDNF) has been shown to play critical roles in neural development, plasticity, and neurodegenerative diseases. The main function of BDNF in the brain is widely accepted to be synaptic regulation. However, how BDNF modulates synaptic transmission, especially the underlying signaling cascades between presynaptic and postsynaptic neurons, remains controversial. In the present study, we investigated the actions of BDNF at rat calyx-type synapses of either sex by measuring the excitatory postsynaptic current (EPSC) and presynaptic calcium current and capacitance changes. We found that BDNF inhibits the EPSC, presynaptic calcium influx, and exocytosis/endocytosis via activation of the presynaptic cannabinoid Type 1 receptors (CB1Rs). Inhibition of the CB1Rs abolished the BDNF-induced presynaptic inhibition, whereas CB1R agonist mimicked the effect of BDNF. Exploring the underlying signaling cascade, we found that BDNF specifically activates the postsynaptic TrkB receptors, inducing the release of endocannabinoids via the PLCγ/DGL pathway and retrogradely activating presynaptic CB1Rs. We also reported the involvement of AC/PKA in modulating vesicle endocytosis, which may account for the BDNF-induced calcium-dependent and -independent regulation of endocytosis. Thus, our study provides new insights into the BDNF/endocannabinoid-associated modulation of neurotransmission in physiological and pathologic processes.

**SIGNIFICANCE STATEMENT** BDNF plays critical roles in the modulation of synaptic strength. However, how BDNF regulates synaptic transmission and its underlying signaling cascade(s) remains elusive. By measuring EPSC and the presynaptic calcium current and capacitance changes at rat calyces, we found that BDNF inhibits synaptic transmission via BDNF-TrkB-eCB signaling pathway. Activation of postsynaptic TrkB receptors induces endocannabinoid release via the PLCγ/DGL pathway, retrogradely activating the presynaptic CB1Rs, inhibiting the AC/PKA, and suppressing calcium influx. Our findings provide a comprehensive understanding of BDNF/endocannabinoid-associated modulation of neuronal activities.

## Abstract (precis)

Brain-derived neurotropic factor (BDNF) has been shown to play critical roles in neural development, plasticity, and neurodegenerative diseases. The main function of BDNF in the brain is widely accepted to be synaptic regulation. However, how BDNF modulates synaptic transmission, especially the underlying signaling cascades between presynaptic and postsynaptic neurons, remains controversial.

## Introduction

Precise and efficient neurotransmission is the basis of neuronal function and plasticity in the CNS (Saheki and De Camilli, 2012; L. G. Wu et al., 2014). Among all neurotrophins, BDNF has attracted much interest for its high expression and potent effects in neural development, functional neural circuit formation, and neurologic diseases (de Jong and Verhage, 2009; Park and Poo, 2013; Choo et al., 2017). BDNF is generally accepted to mainly regulate synaptic function on both excitatory and inhibitory synapses, and one key aspect of the diverse effects of BDNF stems from its complex signaling cascade (Lu et al., 2014). Although many studies have reported BDNF as a potent modulator of synaptic transmission via activation of the tropomyosin receptor kinase B (TrkB) receptor, the underlying signaling cascade is still controversial (Reichardt, 2006; Guo et al., 2018; Lin et al., 2018). A recent study reported that BDNF inhibits synaptic transmission by slowing presynaptic calcium current (ICa) activation, and impairs subsequent exocytosis/endocytosis via activation of the TrkB receptors at a giant calyx-type synapse located in the brainstem (Baydyuk et al., 2015). However, at calyces, the TrkB receptors are expressed not only in the presynaptic nerve terminal, but also in the postsynaptic principal neuron. Whether presynaptic and/or postsynaptic TrkB receptors are involved in the BDNF-induced inhibitory effect is unclear. In addition, the signaling cascade downstream of TrkB activation remains unknown. Interestingly, a previous study reported that BDNF-TrkB signaling in the postsynaptic dendrite leads to a decrease in the probability of presynaptic GABA release on layer 2/3 neocortical inhibitory synapses by rapid mobilization of endocannabinoids (eCBs) into the synaptic cleft (Lemtiri-Chlieh and Levine, 2010). Activation of presynaptic cannabinoid Type 1 receptors (CB1Rs) at the inhibitory synapse makes it necessary to investigate whether BDNF-TrkB signaling at the glutamatergic postsynaptic neuron can also activate the release of eCBs to retrogradely inhibit presynaptic function.

Many studies have demonstrated that eCBs are key activity-dependent signals that can modulate synaptic transmission by activating presynaptic CB1Rs (Castillo et al., 2012). For example, strong depolarization of the postsynaptic neuron can lead to reduced synaptic transmission via the release of eCBs (Wilson and Nicoll, 2001). In addition, excessive glutamate release could activate metabotropic glutamate receptors (mGluRs), promoting the synthesis of eCBs to retrogradely regulate neurotransmission (Kushmerick et al., 2004). Evidence also supports mutual interactions between BDNF and eCB signaling. CB1R antagonist has been shown to block BDNF-induced LTP and LTD (Maglio et al., 2018; Pan et al., 2019). Furthermore, CB1R activation upregulates BDNF expression via the PI3K/Akt/mTORC1 pathway (Blázquez et al., 2015), whereas BDNF can induce the release of eCBs at neocortical inhibitory synapses (Lemtiri-Chlieh and Levine, 2010; Zhao and Levine, 2014) and dopamine neurons in the mouse midbrain (Zhong et al., 2015). A recent study showed that the BDNF-induced increase in the mEPSC frequency can be unmasked by blocking eCB signaling at cortical excitatory synapses, suggesting opposing roles of BDNF (Yeh et al., 2017). Whether such opposing roles of BDNF also exist at the glutamatergic calyx-type synapse is not yet known.

CB1Rs are GPCRs and have a well-documented inhibitory effect on adenylyl cyclase (AC) and protein kinase A (PKA) (Childers and Deadwyler, 1996; Castillo et al., 2012). A previous study demonstrated that the AC/PKA signaling pathway can modulate vesicle endocytosis in an activity-dependent manner (Yao and Sakaba, 2012). These findings urged us to investigate whether AC/PKA signaling is involved in BDNF-induced inhibition of synaptic transmission at calyx of Held synapses.

In the present study, we used time-resolved capacitance measurements at a giant glutamatergic synapse, the calyx of Held, to investigate the signaling cascade underlying BDNF-induced inhibition of synaptic transmission (Barnes-Davies and Forsythe, 1995; Borst et al., 1995). We found that BDNF selectively activates postsynaptic TrkB receptors, which induces the release of eCBs in a calcium-dependent manner and retrogradely activates presynaptic CB1Rs to induce presynaptic inhibition. These results suggest a different interpretation of the previous study (Baydyuk et al., 2015), and the trans-synaptic signaling cascade of BDNF-TrkB-eCB coupling may provide a comprehensive understanding of neurotrophin-regulated neurotransmission in the CNS.

## Materials and Methods

### 

#### 

##### Animals, slice preparation, and electrophysiology

Sprague Dawley rats of either sex were used on postnatal day 8-10 (p8-p10). Brain slices were prepared as described previously (Sun et al., 2016; Liu et al., 2019). Briefly, after the pups were decapitated, and blocks of tissue containing the medial nucleus of the trapezoid body (MNTB) were quickly immersed in low calcium ACSF solution, pH 7.4, containing the following (in mm): 125 NaCl, 25 NaHCO_3_, 3 *myo*-inositol, 2.5 KCl, 1.25 NaH_2_PO_4_, 2 sodium pyruvate, 3 MgCl_2_, 0.05 CaCl_2_, 0.4 ascorbic acid, and 25 glucose. The ACSF solution was bubbled with 95% O_2_/5% CO_2_. Parasagittal brain slices (200-μm-thick) were prepared using a vibratome (VT 1200s, Leica Microsystems) and recovered in normal ACSF with 1 mm Mg^2+^ and 2 mm Ca^2+^ at 37°C bubbled with 95% O_2_/5% CO_2_ for 30 min. All electrophysiological recordings were performed at room temperature (22°C-24°C). The measurements of presynaptic ICa and capacitance changes were performed in a whole-cell configuration using an EPC-10 amplifier (HEKA) with software lock-in amplifier (1000 Hz sine wave, peak-to-peak voltage ≤ 60 mV). The presynaptic recording solution, pH 7.4, contained the following (in mm): 105 NaCl, 25 NaHCO_3_, 3 *myo*-inositol, 2.5 KCl, 1.25 NaH_2_PO_4_, 2 sodium pyruvate, 1 MgCl_2_, 2 CaCl_2_, 0.4 ascorbic acid, 25 glucose, 0.001 TTX, and 20 TEA-Cl. The solution was bubbled with 95% O_2_/5% CO_2_. The presynaptic pipette (3–5 mΩ) solution, pH 7.2 (adjusted with CsOH) contained the following (in mm): 125 Cs-gluconate, 20 CsCl, 4 Mg-ATP, 10 Na_2_-phosphocreatine, 0.3 GTP, 10 HEPES, and 0.05 BAPTA. The series resistance (<10 mΩ) was compensated by 65% (10 μs lag).

For postsynaptic recordings, EPSCs were induced by an afferent stimulus via a bipolar electrode placed near the midline of the MNTB. Stimulation pulses were delivered every 10 s (AM2100, A-M Systems), and the voltage was set to 20% above threshold (<10 V). EPSCs were recorded by an EPC-10 amplifier via a pipette (2-3 mΩ) containing the following (in mm): 125 K-gluconate, 20 KCl, 10 Na_2_-phosphocreatine, 0.3 GTP, 4 Mg-ATP, 10 HEPES, and 0.5 EGTA, pH 7.2 (adjusted with KOH). The series resistance (<10 mΩ) was compensated by 95% (10 μs lag). For paired-pulse recording, a paired stimulus was applied with an interval of 20 ms to induce two consecutive EPSCs. The paired-pulse ratio (PPR) was calculated as the second EPSC divided by the first EPSC (Liu et al., 2019).

BDNF was purchased from Merck and applied in the extracellular recording solution at a final concentration of 100 ng/ml. WIN55212-2, AM251, forskolin, and K252a were purchased from Sigma Millipore. MDL12330A, KT5720, U73122, and RHC80267 were purchased from Tocris Bioscience. All drugs except BDNF were dissolved in DMSO. The final concentration of DMSO was 0.1% (X. S. Wu et al., 2009).

All of the methods were conducted in accordance with the approved guidelines, and all animal experimental protocols were approved by the Animal Care and Use Committee of Fudan University.

##### Immunohistochemistry

The Sprague Dawley rats (p8-p10) were anesthetized using Nembutal and transcardially perfused with 4% PFA (Electron Microscopy Sciences). The brain was immersed in 4% PFA overnight and infiltrated with 20% and 30% sucrose for another 24 h. The brain was embedded in OCT (Electron Microscopy Sciences) and cut in 20-μm-thick slices using a cryostat (CM3050S, Leica Microsystems). Sections containing calyces were permeabilized with 0.5% Triton X-100 and blocked with 5% goat serum. The target proteins were identified using a rabbit antibody against CB1R (1:100; Abcam), a rabbit antibody against TrkB receptor (1:100; Abcam), and a mouse antibody against Bassoon (1:100; Abcam). DyLight-594 donkey anti-rabbit antibody and DyLight-488 donkey anti-mouse antibody (1:100; Thermo Fisher Scientific) were used as secondary antibodies. Images were collected by an LSM700 confocal microscope (Carl Zeiss, 63× oil-immersion objection, 1.3 numerical aperture).

To detect activation of postsynaptic TrkB receptors, parasagittal brain slices (400 μm thick) containing MNTB were prepared and incubated in normal ACSF in the absence or presence of BDNF (100 ng/ml) for 30 min. The target proteins were identified using a mouse antibody against p-Trk (1:50; Santa Cruz Biotechnology) and a rabbit antibody against microtubule-associated protein 2 (MAP2; 1:200; Abcam). DyLight-594 donkey anti-mouse antibody and DyLight-488 donkey anti-rabbit antibody (1:100; Thermo Fisher Scientific) were used as secondary antibodies. ImageJ software (National Institutes of Health) was used to quantify the TrkB activation. The relative fluorescence was calculated as the fluorescence ratio between p-TrkB and MAP2.

##### Data collection and measurements

As described previously (Xue et al., 2012b; Sun et al., 2016), capacitance measurements were made within 10 min after break-in to avoid rundown, and capacitance jumps were measured 250 ms after depolarization to avoid artifacts. The time constant (τ) was obtained from monoexponential or biexponential fitting of the capacitance decay. The initial rate of endocytosis (Rate_endo_) was measured 1-2 s after depolarization. The percentage of residual capacitance 15 (ΔCm_15s_%) or 30 s (ΔCm_30s_%) after depolarization was measured to represent capacitance recovery.

For typical drug bath application experiments, BDNF was applied to the recording chamber by a peristaltic pump at least 30 min before recording at room temperature. When BDNF was coapplied with another drug, both the drug and BDNF were delivered to the chamber at least 30 min before recording and continuously present throughout the experiment. For intracellular drug application experiments, drugs were added to the presynaptic or postsynaptic pipette solution before recording. To specifically block the postsynaptic TrkB receptors, 200 nm K252a was included in the postsynaptic pipette solution via a whole-cell configuration before application of BDNF. After bath application of BDNF for 20 min, we obtained paired recordings by applying another presynaptic pipette at the nerve terminal of the same synapse. The same methods were applied to postsynaptic delivery of BAPTA (20 mm).

##### Experimental design and statistical analyses

For experiments recording the ICa, capacitance changes, and postsynaptic responses, each group of data were collected from 5 to 13 calyces, which were from 3 to 7 rats of either sex. All data are presented as mean ± SEM. We used the Kolmogorov-Smirnov test to assess differences in calcium inactivation and paired or unpaired Student's *t* test to assess differences between two groups. One-way ANOVA with *post hoc* Bonferroni test was applied for multiple group comparisons. A *p* value < 0.05 was considered significant. All statistical analyses were performed using MATLAB (2019b, The MathWorks). Details of all statistical analysis are provided in Table 1.

**Table 1.** Statistical analysis per figure

Figure	Test	*Post hoc* comparison
1*B*	Repeated-measures one-way ANOVA EPSC: *F*_(1.557,10.90)_ = 12.90, *p* = 0.0021 PPR: *F*_(1.693,11.85)_ = 38.98, *p* < 0.0001	Bonferroni's multiple comparisons test EPSC: Ctrl vs BDNF, *p* = 0.0020; BDNF vs Washout, *p* = 0.0218 PPR: Ctrl vs BDNF, *p* = 0.0006; BDNF vs Washout, *p* = 0.0007
1*C*	Unpaired Student's *t* test (two-tailed) ICa: *t* = 7.851, df = 13, *p* < 0.0001; Rise time: *t* = 0.5952, df = 13, *p* = 0.5619	
1*D*	Unpaired Student's *t* test (two-tailed)	
	−10 mV: *t* = 3.233, df = 9, *p* = 0.0103;	
	0 mV: *t* = 2.750, df = 9, *p* = 0.0225;	
	10 mV: *t* = 2.809, df = 9, 0.0204;	
	20 mV: *t* = 2.798, df = 9, 0.0208;	
	30 mV: *t* = 2.627, df = 9, 0.0275;	
	40 mV: *t* = 2.594, df = 9, 0.0290	
1*E*	Kolmogorov-Smirnov test *p* = 0.9819	
1*G*	Unpaired Student's *t* test (two-tailed)	
	ΔCm_1ms_: *t* = 3.258, df = 9, *p* = 0.0099;	
	ΔCm_2ms_: *t* = 2.483, df = 9, *p* = 0.0348;	
	ΔCm_5ms_: *t* = 4.480, df = 9, *p* = 0.0015;	
	ΔCm_10ms_: *t* = 4.181, df = 9, *p* = 0.0024;	
	ΔCm_20ms_: *t* = 3.958, df = 9, *p* = 0.0033;	
	ΔCm_30ms_: *t* = 1.630, df = 9, *p* = 0.1375;	
	ΔCm_50ms_: *t* = 0.02962, df = 9, *p* = 0.9770	
	Probability: *t* = 3.999, df = 9, *p* = 0.0031	
2*D*	One-way ANOVA	Bonferroni's multiple comparisons test
	ΔCm: *F*_(7,50)_ = 7.459, *p* < 0.0001	ΔCm: Ctrl vs BDNF, *p* = 0.0017; Ctrl vs WIN, *p* = 0.0004; BDNF vs ANA+BDNF, *p* = 0.0015; BDNF vs AM251+BDNF, *p* = 0.0369; WIN vs AM251+WIN, *p* = 0.0065
	ICa: *F*_(7,50)_ = 8.813, *p* < 0.0001	ICa: Ctrl vs BDNF, *p* = 0.0037; Ctrl vs WIN, *p* = 0.0001; BDNF vs ANA+BDNF, *p* = 0.0019; BDNF vs AM251+BDNF, *p* = 0.0076; WIN vs AM251+WIN, *p* = 0.0018
	Rate_endo_: *F*_(7,50)_ = 8.620, *p* < 0.0001	Rate_endo_: Ctrl vs BDNF, *p* = 0.0032; Ctrl vs WIN, *p* = 0.0009; BDNF vs ANA+BDNF, *p* = 0.0073; BDNF vs AM251+BDNF, *p* = 0.0318; WIN vs WIN+AM251, *p* = 0.0091
	ΔCm_15s_%: *F*_(7,50)_ = 6.459, *p* < 0.0001	ΔCm_15s_%: Ctrl vs BDNF, *p* = 0.0002; Ctrl vs WIN, *p* = 0.0140; BDNF vs ANA+BDNF, *p* = 0.0167; BDNF vs AM251+BDNF, *p* = 0.0126; WIN vs AM251+WIN, *p* = 0.0271
2*E*	One-way ANOVA	Bonferroni's multiple comparisons test
	ICa: *F*_(2,16)_ = 10.80, *p* = 0.0011	K252a_post_ vs BDNF, *p* = 0.0130; BDNF vs K252a_post_+BDNF, *p* = 0.0013
2*F*	One-way ANOVA	Bonferroni's multiple comparisons test
	ΔCm: *F*_(2,16)_ = 12.78, *p* = 0.0005	ΔCm: K252a_post_ vs BDNF, *p* = 0.0018; BDNF vs K252a_post_+BDNF, *p* = 0.0014
	Rate_endo_: *F*_(2,16)_ = 13.64, *p* = 0.0003	Rate_endo_: K252a_post_ vs BDNF, *p* = 0.0026; BDNF vs K252a_post_+BDNF, *p* = 0.0006
	ΔCm_15s_%: *F*_(2,16)_ = 5.362, *p* = 0.0165	ΔCm_15s_%: K252a_post_ vs BDNF, *p* = 0.0487; BDNF vs K252a_post_+BDNF, *p* = 0.0318
3*B*	One-way ANOVA	Bonferroni's multiple comparisons test
	ΔCm: *F*_(7,62)_ = 7.346, *p* < 0.0001	ΔCm: Ctrl vs BDNF, *p* = 0.0021; Ctrl vs WIN, *p* = 0.0025; BDNF vs ANA+BDNF, *p* = 0.0321; BDNF vs AM251+BDNF, *p* = 0.0051; WIN vs AM251+WIN, *p* = 0.0054
	QICa: *F*_(7,62)_ = 8.623, *p* < 0.0001	QICa: Ctrl vs BDNF, *p* = 0.0002; Ctrl vs WIN, *p* = 0.0003; BDNF vs ANA+BDNF, *p* = 0.0059; BDNF vs AM251+BDNF, *p* = 0.0004; WIN vs AM251+WIN, *p* = 0.0153
	Rate_endo_: *F*_(7,62)_ = 17.11, *p* < 0.0001	Rate_endo_: Ctrl vs BDNF, *p* < 0.0001; Ctrl vs WIN, *p* < 0.0001; BDNF vs ANA+BDNF, *p* < 0.0001; BDNF vs AM251+BDNF, *p* < 0.0001; WIN vs AM251+WIN, *p* = 0.0004
	ΔCm_30s_%: *F*_(7,62)_ = 6.363, *p* < 0.0001	ΔCm_30s_%: Ctrl vs BDNF, *p* = 0.0025; Ctrl vs WIN, *p* = 0.0119; BDNF vs ANA+BDNF, *p* = 0.0227; BDNF vs AM251+BDNF, *p* = 0.0231; WIN vs AM251+WIN, *p* = 0.0255
3*C*	One-way ANOVA	Bonferroni's multiple comparisons test
	QICa: *F*_(2,19)_ = 10.01, *p* = 0.0011	K252a_post_ vs BDNF, *p* = 0.0183; BDNF vs K252a_post_+BDNF, *p* = 0.0010
3*D*	One-way ANOVA	Bonferroni's multiple comparisons test
	ΔCm: *F*_(2,19)_ = 4.853, *p* = 0.0198	ΔCm: K252a_post_ vs BDNF, *p* = 0.0438; BDNF vs K252a_post_+BDNF, *p* = 0.0402
	Rate_endo_: *F*_(2,19)_ = 14.97, *p* = 0.0001	Rate_endo_: K252a_post_ vs BDNF, *p* = 0.0007; BDNF vs K252a_post_+BDNF, *p* = 0.0003
	ΔCm_30s_%: *F*_(2,19)_ = 23.39, *p* < 0.0001	ΔCm_30s_%: K252a_post_ vs BDNF, *p* < 0.0001; BDNF vs K252a_post_+BDNF, *p* < 0.0001
4	Unpaired Student's *t* test (two-tailed)	
	*t* = 9.838, df = 24, *p* < 0.0001	
5*B*	Paired Student's *t* test (two-tailed)	
	EPSC: *t* = 0.1761, df = 4, *p* = 0.8688; PPR: *t* = 1.430, df = 4, *p* = 0.2259	
5*C*	One-way ANOVA	Bonferroni's multiple comparisons test
	ICa: *F*_(2,14)_ = 13.26, *p* = 0.0006	Vehicle vs Vehicle+BDNF, *p* = 0.0010; Vehicle+BDNF vs BAPTA+BDNF, *p* = 0.0022
5*D*	One-way ANOVA	Bonferroni's multiple comparisons test
	ΔCm: *F*_(2,14)_ = 6.408, *p* = 0.0106	ΔCm: Vehicle vs Vehicle+BDNF, *p* = 0.0173; Vehicle+BDNF vs BAPTA+BDNF, *p* = 0.0271
	Rate_endo_: *F*_(2,14)_ = 8.801, *p* = 0.0033	Rate_endo_: Vehicle vs Vehicle+BDNF, *p* = 0.0067; Vehicle+BDNF vs BAPTA+BDNF, *p* = 0.0080
	ΔCm_15s_%: *F*_(2,14)_ = 13.27, *p* = 0.0006	ΔCm_15s_%: Vehicle vs Vehicle+BDNF, *p* = 0.0010; Vehicle+BDNF vs BAPTA+BDNF, *p* = 0.0021
5*E*	One-way ANOVA	Bonferroni's multiple comparisons test
	QICa: *F*_(2,14)_ = 23.81, *p* < 0.0001	Vehicle vs Vehicle+BDNF, *p* < 0.0001; Vehicle+BDNF vs BAPTA+BDNF, *p* = 0.0002
5*F*	One-way ANOVA	Bonferroni's multiple comparisons test
	ΔCm: *F*_(2,14)_ = 10.68, *p* = 0.0015	ΔCm: Vehicle vs Vehicle+BDNF, *p* = 0.0034; Vehicle+BDNF vs BAPTA+BDNF, *p* = 0.0052
	Rate_endo_: *F*_(2,14)_ = 21.21, *p* < 0.0001	Rate_endo_: Vehicle vs Vehicle+BDNF, *p* = 0.0002; Vehicle+BDNF vs BAPTA+BDNF, *p* = 0.0002
	ΔCm_30s_%: *F*_(2,14)_ = 36.09, *p* < 0.0001	ΔCm_30s_%: Vehicle vs Vehicle+BDNF, *p* < 0.0001; Vehicle+BDNF vs BAPTA+BDNF, *p* < 0.0001
6*B*	One-way ANOVA	Bonferroni's multiple comparisons test
	ΔCm: *F*_(5,33)_ = 5.803, *p* = 0.0006	ΔCm: DMSO_bath_ vs BDNF, *p* = 0.0003; BDNF vs U73122+BDNF, *p* = 0.0122; BDNF vs RHC+BDNF, *p* = 0.0331
	ICa: *F*_(5,33)_ = 5.253, *p* = 0.0012	ICa: DMSO_bath_ vs BDNF, *p* = 0.0042; BDNF vs U73122+BDNF, *p* = 0.0181; BDNF vs RHC+BDNF, *p* = 0.0490
	Rate_endo_: *F*_(5,33)_ = 4.555, *p* = 0.0029	Rate_endo_: DMSO_bath_ vs BDNF, *p* = 0.0050; BDNF vs U73122+BDNF, *p* = 0.0430; BDNF vs RHC+BDNF, *p* = 0.0195
	ΔCm_15s_%: *F*_(5,33)_ = 9.312, *p* < 0.0001	ΔCm_15s_%: DMSO_bath_ vs BDNF, *p* < 0.0001; BDNF vs U73122+BDNF, *p* = 0.0002; BDNF vs RHC+BDNF, *p* = 0.0009
6*D*	One-way ANOVA	Bonferroni's multiple comparisons test
	ΔCm: *F*_(5,36)_ = 7.033, *p* = 0.0001	ΔCm: DMSO_bath_ vs BDNF, *p* = 0.0031; BDNF vs U73122+BDNF, *p* = 0.0194; BDNF vs RHC+BDNF, *p* = 0.0476
	QICa: *F*_(5,36)_ = 6.087, *p* = 0.0004	QICa: DMSO_bath_ vs BDNF, *p* = 0.0159; BDNF vs U73122+BDNF, *p* = 0.0280; BDNF vs RHC+BDNF, *p* = 0.0052
	Rate_endo_: *F*_(5,36)_ = 16.01, *p* < 0.0001	Rate_endo_: DMSO_bath_ vs BDNF, *p* < 0.0001; BDNF vs U73122+BDNF, *p* < 0.0001; BDNF vs RHC+BDNF, *p* = 0.0001
	ΔCm_30s_%: *F*_(5,36)_ = 7.178, *p* < 0.0001	ΔCm_30s_%: DMSO_bath_ vs BDNF, *p* = 0.0051; BDNF vs U73122+BDNF, *p* = 0.0261; BDNF vs RHC+BDNF, *p* = 0.0215
7*A*	Unpaired Student's *t* test (two-tailed) ICa: *t* = 0.4859, df = 13, *p* = 0.6351	
7*B*	Unpaired Student's *t* test (two-tailed)	
	ΔCm: *t* = 0.7345, df = 13, *p* = 0.4757	
	Rate_endo_: *t* = 0.4740, df = 13, *p* = 0.6434	
	ΔCm_15s_%: *t* = 1.228, df = 13, *p* = 02413	
7*C*	Unpaired Student's *t* test (two-tailed) QICa: *t* = 0.2790, df = 14, *p* = 0.7844	
7*D*	Unpaired Student's *t* test (two-tailed)	
	ΔCm: *t* = 0.5201, df = 14, *p* = 0.6111	
	Rate_endo_: *t* = 3.608, df = 14, *p* = 0.0029	
	ΔCm_30s_%: *t* = 4.246, df = 14, *p* = 0.0008	
7*E*	Unpaired Student's *t* test (two-tailed) QICa: *t* = 0.2457, df = 12, *p* = 0.8101	
7*F*	Unpaired Student's *t* test (two-tailed)	
	ΔCm: *t* = 0.2279, df = 12, *p* = 0.8235	
	Rate_endo_: *t* = 3.985, df = 12, *p* = 0.0018	
	ΔCm_30s_%: *t* = 4.786, df = 12, *p* = 0.0004	
8*B*	One-way ANOVA	Bonferroni's multiple comparisons test
	ΔCm: *F*_(7,44)_ = 8.465, *p* < 0.0001	ΔCm: DMSO_pre_ vs WIN, *p* = 0.0206; DMSO_pre_ vs MDL, *p* = 0.0155; DMSO_pre_ vs KT, *p* = 0.0351; DMSO_pre_ vs MDL+WIN, *p* = 0.0071; DMSO_pre_ vs KT+WIN, *p* = 0.0140; WIN vs Forskolin+WIN, *p* = 0.0103
	ICa: *F*_(7,44)_ = 11.99, *p* < 0.0001	ICa: DMSO_pre_ vs WIN, *p* = 0.0010; DMSO_pre_ vs MDL, *p* = 0.0002; DMSO_pre_ vs KT, *p* < 0.0001; DMSO_pre_ vs MDL+WIN, *p* = 0.0001; DMSO_pre_ vs KT+WIN, *p* = 0.0001; WIN vs Forskolin+WIN, *p* = 0.0019
	Rate_endo_: *F*_(7,44)_ = 9.960, *p* < 0.0001	Rate_endo_: DMSO_pre_ vs WIN, *p* = 0.0189; DMSO_pre_ vs MDL, *p* = 0.0049; DMSO_pre_ vs KT, *p* = 0.0187; DMSO_pre_ vs MDL+WIN, *p* = 0.0075; DMSO_pre_ vs KT+WIN, *p* = 0.0068; WIN vs Forskolin+WIN, *p* = 0.0055
	ΔCm_15s_%: *F*_(7,44)_ = 8.221, *p* < 0.0001	ΔCm_15s_%: DMSO_pre_ vs WIN, *p* = 0.0069; DMSO_pre_ vs MDL, *p* = 0.0009; DMSO_pre_ vs KT, *p* = 0.0008; DMSO_pre_ vs MDL+WIN, *p* = 0.0470; DMSO_pre_ vs KT+WIN, *p* = 0.0107; WIN vs Forskolin+WIN, *p* = 0.0476
8*D*	One-way ANOVA	Bonferroni's multiple comparisons test
	ΔCm: *F*_(9,60)_ = 8.949, *p* < 0.0001	ΔCm: DMSO_pre_ vs WIN, *p* = 0.0005; DMSO_pre_ vs MDL, *p* = 0.0017; DMSO_pre_ vs KT, *p* = 0.0012; DMSO_pre_ vs MDL+WIN, *p* = 0.0003; DMSO_pre_ vs KT+WIN, *p* = 0.0005; WIN vs Forskolin+WIN, *p* = 0.0013
	QICa: *F*_(9,60)_ = 10.41, *p* < 0.0001	QICa: DMSO_pre_ vs WIN, *p* = 0.0167; DMSO_pre_ vs MDL, *p* < 0.0001; DMSO_pre_ vs KT, *p* = 0.0007; DMSO_pre_ vs MDL+WIN, *p* = 0.0007; DMSO_pre_ vs KT+WIN, *p* = 0.0039; WIN vs Forskolin+WIN, *p* = 0.0425
	Rate_endo_: *F*_(9,60)_ = 15.33, *p* < 0.0001	Rate_endo_: DMSO_pre_ vs WIN, *p* < 0.0001; DMSO_pre_ vs MDL, *p* < 0.0001; DMSO_pre_ vs KT, *p* < 0.0001; DMSO_pre_ vs MDL+WIN, *p* < 0.0001; DMSO_pre_ vs KT+WIN, *p* < 0.0001; WIN vs Forskolin+WIN, *p* = 0.0003
	ΔCm_30s_%: *F*_(9,60)_ = 7.649, *p* < 0.0001	ΔCm_30s_%: DMSO_pre_ vs WIN, *p* = 0.0001; DMSO_pre_ vs MDL, *p* = 0.0016; DMSO_pre_ vs KT, *p* = 0.0002; DMSO_pre_ vs MDL+WIN, *p* = 0.0014; DMSO_pre_ vs KT+WIN, *p* = 0.0045; WIN vs Forskolin+WIN, *p* = 0.0023
8*E*	One-way ANOVA (Ctrl_2Ca_ is identical to Ctrl in Fig. 8*C*, and the multiple comparison was made among MDL_3.5Ca_, KT_3.5Ca_, and other eight groups in Fig. 8*D*)	Bonferroni's multiple comparisons test
	QICa: *F*_(9,60)_ = 10.41, *p* < 0.0001	QICa: Ctrl_2Ca_ vs MDL_3.5Ca_, *p* > 0.9999; Ctrl_2Ca_ vs KT_3.5Ca_, *p* > 0.9999
8*F*	One-way ANOVA (Ctrl_2Ca_ is identical to Ctrl in Fig. 8*C*, and the multiple comparison was made among MDL_3.5Ca_, KT_3.5Ca_, and other eight groups in Fig. 8*D*)	Bonferroni's multiple comparisons test
	ΔCm: *F*_(9,60)_ = 8.949, *p* < 0.0001	ΔCm: Ctrl_2Ca_ vs MDL_3.5Ca_, *p* > 0.9999; Ctrl_2Ca_ vs KT_3.5Ca_, *p* > 0.9999
	Rate_endo_: *F*_(9,60)_ = 15.33, *p* < 0.0001	Rate_endo_: Ctrl_2Ca_ vs MDL_3.5Ca_, *p* = 0.0002; Ctrl_2Ca_ vs KT_3.5Ca_, *p* = 0.0011
	ΔCm_30s_%: *F*_(9,60)_ = 7.649, *p* < 0.0001	ΔCm_30s_%: Ctrl_2Ca_ vs MDL_3.5Ca_, *p* = 0.0332; Ctrl_2Ca_ vs KT_3.5Ca_, *p* = 0.0456

## Results

### BDNF inhibits apparent EPSC, presynaptic ICa, and release probability

**Figure 1.**
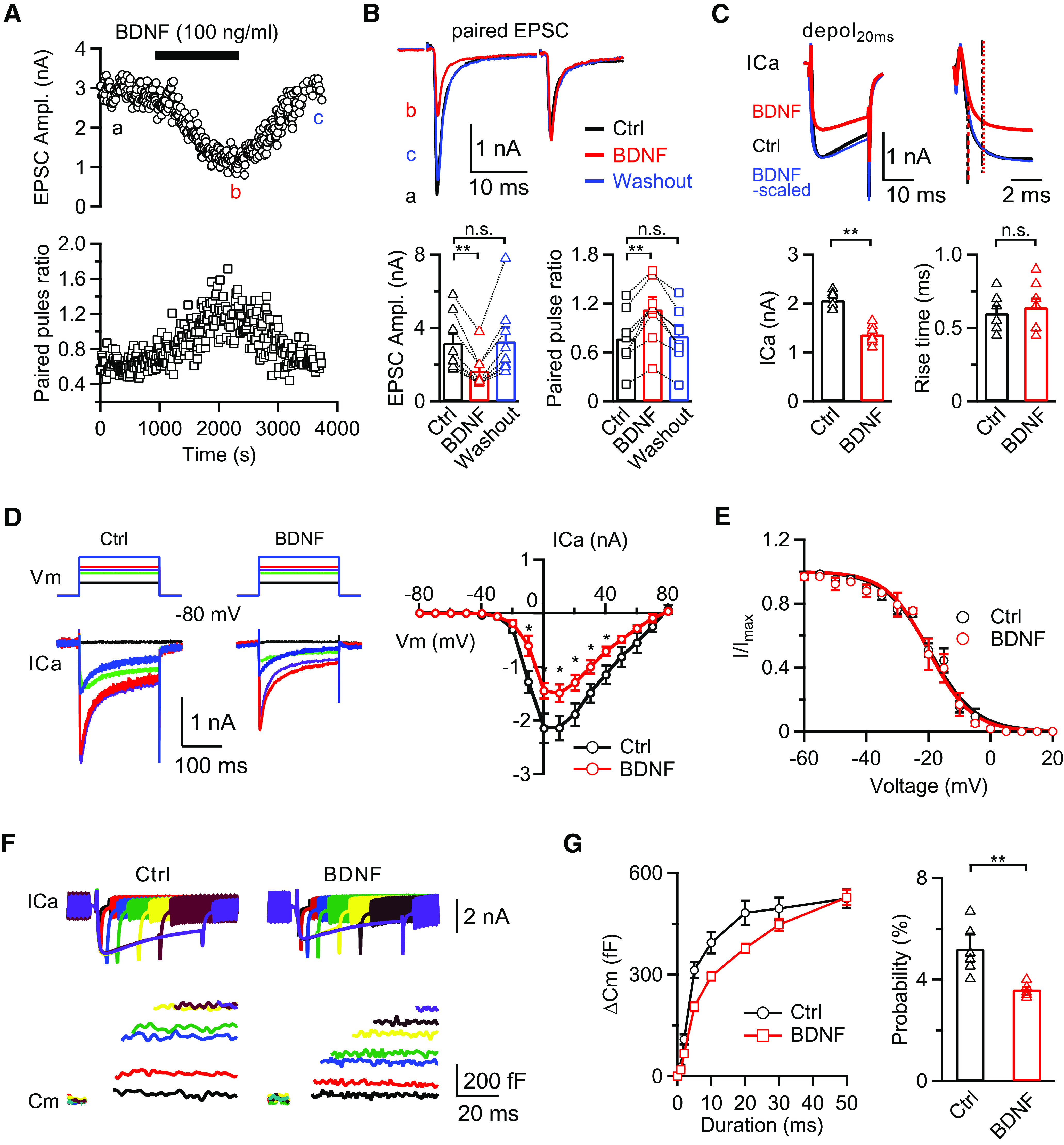
BDNF inhibits EPSC, presynaptic ICa, and release probability. ***A***, Top, Sampled paired EPSC recordings in response to 0.1 Hz fiber stimulation at the midline of the trapezoid body; 100 ng/ml BDNF was added to the extracellular solution after obtaining a baseline, and BDNF was washed out after the reduced EPSC amplitudes were stable. Bottom, The corresponding PPR calculated from the paired EPSC amplitudes. ***B***, Top, Sampled EPSC pairs at time points a (baseline, black), b (BDNF treatment, red), and c (washout, blue) from ***A*** were overlapped, showing the EPSC changes in response to BDNF application. Bottom, Statistics for EPSC amplitude and PPR (*n* = 8) from ***A*** using a repeated-measures one-way ANOVA with Bonferroni's multiple comparisons test. **p < 0.01. ***C***, Top, Averaged traces of ICa induced by depol_20ms_ in the control group (*n* = 7; black) and in the presence of BDNF (100 ng/ml, *n* = 8; red). ICa of the BDNF-treated group is scaled for comparison (BDNF-scaled, blue). Dashed lines indicate the 20% and 80% rise time. Bottom, Statistics for ICa amplitude and the 20%-80% rise time induced by depol_20ms_ using an unpaired Student's *t* test. **p < 0.01. ***D***, Left, Sampled ICa traces in response to 200 ms depolarization pulses from −80 to −40 mV (black), −10 (green), 0 (purple), 10 (red), and 40 mV (blue) in the control and BDNF-treated groups. Right, Plot of the current–voltage relationship in control (*n* = 5 for each data point; black) and BDNF-treated calyces (*n* = 6 for each data point; red). *p* values were calculated using an unpaired Student's *t* test. *p < 0.05. ***E***, Plot of ICa inactivation curves in the control (*n* = 5 for each data point; black) and BDNF-treated calyces (*n* = 5 for each data point; red). ***F***, Sampled ICa (top) and capacitance changes (bottom) induced by 1 (black), 2 (red), 5 (blue), 10 (green), 20 (yellow), 30 (brown), and 50 ms (purple) depolarization pulses from −80 to 10 mV in the control and BDNF-treated groups. ***G***, Left, The relationship between ΔCm and the duration of depolarization pulses in the control (*n* = 6 for each data point; black) and BDNF-treated (*n* = 5 for each data point; red) groups. Right, Statistics of the release probability measured by the percentage of RRP release induced by a 1 ms depolarization pulse from −80 to 10 mV in the control (*n* = 6; black) and BDNF-treated groups (*n* = 5; red) using an unpaired Student's *t* test. Data were from left. **p < 0.01. n.s., not significant. Detailed statistical information is provided in Table 1.

First, we investigated how BDNF modulates synaptic transmission by examining the EPSCs of the postsynaptic principal neurons at rat calyces in a whole-cell configuration with 0.5 mm EGTA in the pipette solution. We applied a pair of stimuli with an interval of 20 ms every 10 s near the midline of the MNTB in p8–p10 rats, which can induce two consecutive EPSCs. The PPR was used to evaluate the change in release probability as described above. Recording a baseline for ∼10 min, the averaged EPSC amplitude was 3.2 ± 0.5 nA (*n* = 8 calyces). After bath application of BDNF (100 ng/ml), the EPSC gradually decreased and reached a plateau in ∼15-20 min (Fig. 1*A*, top). The EPSC amplitude decreased ∼50% (1.6 ± 0.3 nA, *n* = 8; Fig. 1*B*) and was significantly smaller than the baseline (*p* = 0.0020, repeated-measures one-way ANOVA with Bonferroni *post hoc* test). The reduced EPSCs recovered to a level similar to baseline after an ∼15–30 min washout (99 ± 7%; *p* > 0.9999, repeated-measures one-way ANOVA with Bonferroni *post hoc* test), showing a reversible inhibitory effect of BDNF (Fig. 1*A*,*B*). The PPR at baseline was 0.77 ± 0.13 and increased to 1.13 ± 0.15 after BDNF application (*p* = 0.0006, repeated-measures one-way ANOVA with Bonferroni *post hoc* test; *n* = 8; Fig. 1*A*, bottom, *B*), suggesting a presynaptic mechanism for BDNF-inhibited synaptic transmission (Liu et al., 2019).

A recent study reported that BDNF slows presynaptic ICa activation to inhibit EPSCs at the rat calyx synapse (Baydyuk et al., 2015). Therefore, we applied a similar 20 ms depolarization pulse from −80 to 10 mV (depol_20ms_) and recorded the ICa at the presynaptic nerve terminal of rat calyces (Fig. 1*C*). We observed an obvious reduction in ICa amplitude after bath application of BDNF (100 ng/ml; Ctrl: 2.1 ± 0.1 nA, *n* = 7; BDNF: 1.4 ± 0.1 nA, *n* = 8; *p* < 0.0001, unpaired *t* test; Fig. 1*C*). However, we did not find a slowing of ICa activation. The 20%–80% rise time for ICa after depol_20ms_ in control rats was 0.60 ± 0.05 ms (*n* = 7), which is similar to the previous report (Baydyuk et al., 2015). After incubation with BDNF (100 ng/ml) in the extracellular solution for 30 min, the 20%-80% rise time was not significantly changed (0.64 ± 0.06 ms, *n* = 8; *p* = 0.5619, unpaired *t* test; Fig. 1*C*). Next, we plotted the current–voltage curve induced by a 200 ms depolarization pulse from −80 mV to −70, −60, … 80 mV with an interval of 30 s (Fig. 1*D*, left); the ICa in the presence of BDNF was smaller than control at every voltage step, but the peak amplitude did not shift (Ctrl: *n* = 5; BDNF: *n* = 6; Fig. 1*D*, right), confirming that BDNF inhibits ICa amplitude but does not affect its activation. We also examined the inactivation curve of the ICa and found no significant difference between the control and BDNF-treated groups (*p* = 0.9819, Kolmogorov-Smirnov test; *n* = 5; Fig. 1*E*). These results suggest that inhibition of the presynaptic ICa amplitude, not a slowdown of ICa activation, is involved in the BDNF-induced inhibition of EPSC amplitude.

The increased PPR recorded for the EPSC after bath application of BDNF suggests a reduction in release probability (Fig. 1*A*,*B*). To address this issue at the presynaptic site, we applied stimulation pulses of various lengths (1, 2, 5, 10, 20, 30, and 50 ms) from −80 to 10 mV to induce vesicle release and determined the changes in the readily releasable pool (RRP) size and release probability in the presence of BDNF as described previously (Xue et al., 2012b). In control rats, depol_20ms_ induced a capacitance jump of 493 ± 35 fF (*n* = 6), which represents the RRP size (Sun and Wu, 2001). Depolarization pulses of 1, 2, 5, and 10 ms induced 6 ± 1%, 23 ± 4%, 65 ± 4%, and 87 ± 5%, respectively, of the capacitance jump induced by depol_20ms_ measured at the same synapses (*n* = 6 for each depolarization step; Fig. 1*F*, left, *G*, black). No further capacitance increase was observed in the control group when the step duration was increased after 30 ms, which is consistent with previous studies showing that a 10–20 ms depolarization pulse from −80 to 10 mV can deplete the RRP (Sun and Wu, 2001; Xue et al., 2012a). After application of BDNF (100 ng/ml) to the extracellular solution, depol_20ms_ induced a capacitance increase of 378 ± 13 fF (*n* = 5), which is smaller than the capacitance jump measured in controls (*p* = 0.0033, unpaired *t* test). When longer stimulation pulses (30–50 ms) were applied, the capacitance jump increased further, until reaching a similar level as controls (528 ± 23 fF after 50 ms depolarization pulse, *n* = 5; *p* = 0.9770, unpaired *t* test; Fig. 1*G*, red), suggesting that the RRP size is not affected. However, depolarization pulses of 1, 2, 5, and 10 ms induced 4 ± 1%, 13 ± 2%, 39 ± 4%, and 56 ± 3%, respectively, of the capacitance jump induced by depol_50ms_ after incubation with BDNF (*n* = 5 for each depolarization step, Fig. 1*F*, right, *G*, red), which is much smaller than the capacitance jump in controls. These results clearly indicate a reduction in the vesicle release probability, which was calculated the percentage of RRP release (ΔCm_1ms_/RRP) induced by an action potential-like stimulation (depol_1ms_, 1 ms depolarization pulse from −80 to 10 mV, the data for depol_1ms_ were obtained from the same experiments; Fig. 1*G*) (Sun and Wu, 2001).

### BDNF induces presynaptic inhibition via activation of postsynaptic TrkB receptor and presynaptic CB1R

**Figure 2.**
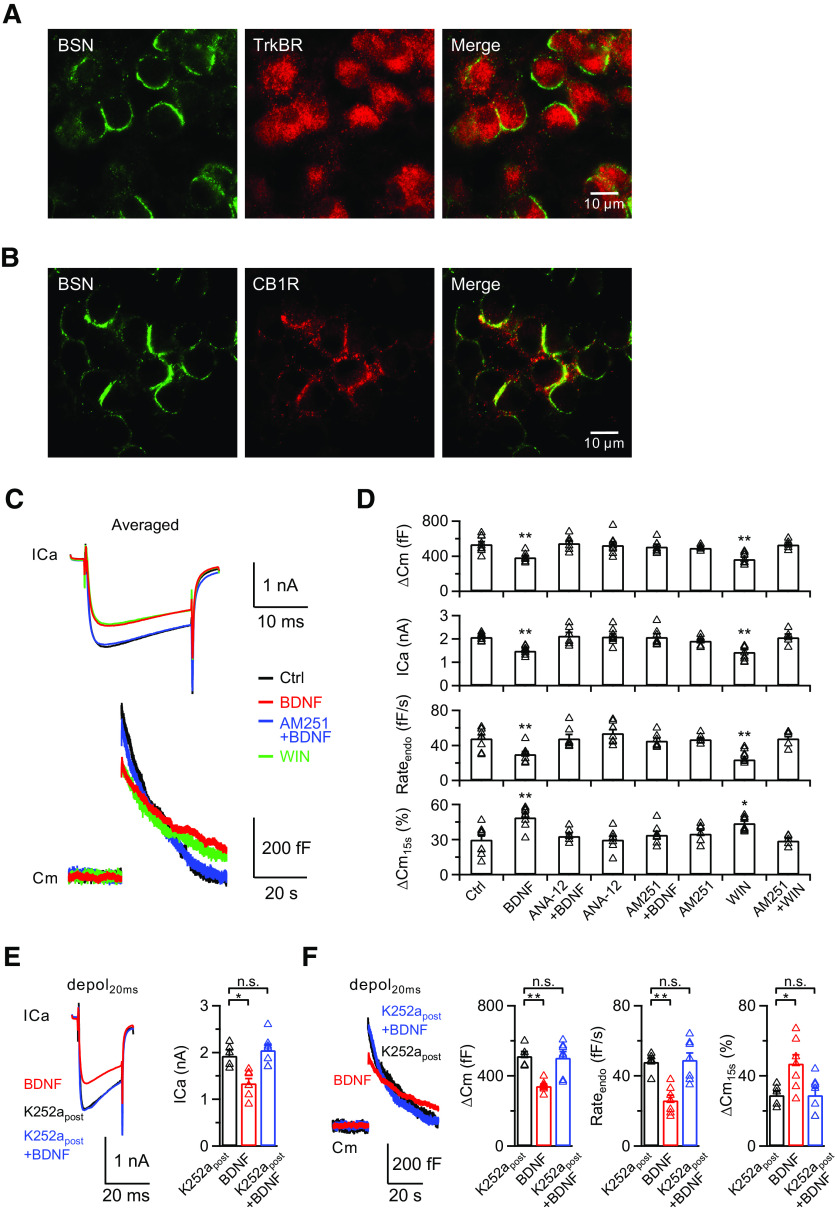
BDNF inhibits presynaptic ICa and exocytosis/endocytosis via activation of postsynaptic TrkB receptors and presynaptic CB1Rs. ***A***, Immunostaining of presynaptic cytomatrix (Bassoon, green) and TrkB receptors (red) at calyces. Scale bar, 10 μm. ***B***, Immunostaining of presynaptic cytomatrix (Bassoon, green) and CB1Rs (red) at calyces. Scale bar, 10 μm. ***C***, Averaged presynaptic ICa (top) and Cm (bottom) induced by depol_20ms_ in the control (Ctrl, black), BDNF treatment (BDNF, red), BDNF treatment in the presence of AM251 (AM251+BDNF, blue), and WIN55212-2 treatment (WIN, green) groups. ***D***, Statistics for ΔCm, ICa, Rate_endo_, and ΔCm_15s_% from different treatments in extracellular solution (Ctrl, *n* = 9; BDNF, *n* = 8; ANA-12+BDNF, *n* = 7; ANA-12, *n* = 8; AM251+BDNF, *n* = 7; AM251, *n* = 6; WIN, *n* = 8; AM251+WIN, *n* = 5). *p* values were calculated using a one-way ANOVA with Bonferroni's multiple comparisons test. *p < 0.05. **p < 0.01. ***E***, Left, Averaged presynaptic ICa induced by depol_20ms_ in the control (K252a_post_, 200 nm K252a in the postsynaptic pipette solution, *n* = 5; black), BDNF treatment (BDNF, without K252a in the postsynaptic pipette solution in the presence of BDNF, *n* = 7; red), and BDNF treatment with K252a (K252a_post_+BDNF, 200 nm K252a in the postsynaptic pipette solution in the presence of BDNF, *n* = 7; blue) groups. All recordings are made in the paired-recording mode. Right, Statistics for ICa in all three groups using a one-way ANOVA with Bonferroni's multiple comparisons test. *p < 0.05. ***F***, Left, Averaged Cm induced by depol_20ms_ from ***E***. Right, Statistics for ΔCm, Rate_endo_, and ΔCm_15s_% in all three groups using a one-way ANOVA with Bonferroni's multiple comparisons test. *p < 0.05. **p < 0.01. n.s., not significant. Detailed statistical information is provided in Table 1.

BDNF regulates synaptic functions via the activation of TrkB receptors, the major BDNF receptors in the CNS (Reichardt, 2006). We examined the location of TrkB receptors at the calyx of Held synapse by immunostaining. The antibody against Bassoon (BSN), a presynaptic cytomatrix protein selectively localized at the active zone of the nerve terminal (tom Dieck et al., 1998), overlapped partially with the staining of the antibody against TrkB receptors (Fig. 2*A*). Staining of TrkB receptors was also observed at the postsynaptic principal neurons, indicating that the TrkB receptors have both presynaptic and postsynaptic expression. We asked whether presynaptic and/or postsynaptic TrkB receptors are involved in the BDNF-induced inhibition of synaptic transmission. A previous study demonstrated that BDNF can act on postsynaptic TrkB receptors to activate the release of eCBs, which would then diffuse retrogradely to reduce the presynaptic release probability of neurotransmitters at cortical inhibitory synapses (Lemtiri-Chlieh and Levine, 2010). In the glutamatergic calyx-type synapse, eCBs are synthesized postsynaptically, and the CB1Rs are specifically localized at the presynaptic nerve terminal (Kushmerick et al., 2004; Zou and Kumar, 2018) (Fig. 2*B*). Therefore, the postsynaptic expression of TrkB receptors, together with the presynaptic localization of CB1Rs, prompted reinvestigation of the previous study to examine the possibility of BDNF-TrkB-CB1R-induced inhibition of synaptic transmission at calyces (Baydyuk et al., 2015).

First, we investigated whether BDNF-induced reduction of presynaptic ICa can inhibit presynaptic vesicle exocytosis and endocytosis at the calyx of Held synapse reported previously (Baydyuk et al., 2015). In control p8-p10 rats, depol_20ms_ induced a calcium influx (2.1 ± 0.1 nA, *n* = 9; Fig. 2*C*, top, black, *D*) and a capacitance jump representing exocytosis (ΔCm; 538 ± 30 fF, *n* = 9; Fig. 2*C*, bottom, black, *D*). The immediately subsequent capacitance decay reflecting endocytosis could be fitted monoexponentially with a time constant of 16.9 ± 1.6 s. The Rate_endo_ measured 1–2 s after depol_20ms_ was 48 ± 4 fF/s (*n* = 9; Fig. 2*C*, bottom, black), which demonstrates slow endocytosis (W. Wu et al., 2005; Xue et al., 2012b). In the presence of BDNF (100 ng/ml) in the extracellular solution, depol_20ms_ induced a ICa of 1.5 ± 0.1 nA and a ΔCm of 387 ± 18 fF (*n* = 8), both of which were significantly lower than control (ICa: *p* = 0.0037; ΔCm: *p* = 0.0017; one-way ANOVA with Bonferroni *post hoc* test; Fig. 2*C*, red, *D*). The Rate_endo_ was also reduced (30 ± 3 fF/s, *n* = 8; *p* = 0.0032, one-way ANOVA with Bonferroni *post hoc* test; Fig. 2*D*), suggesting inhibition in slow endocytosis. The capacitance decay did not return to baseline within 30 s, which is difficult to fit monoexponentially. The remaining ΔCm 15 s after depol_20ms_ was 49 ± 3% (ΔCm_15s_%; *n* = 8; Fig. 2*D*) of the maximum ΔCm after depol_20ms_, which is still remarkably higher than control (30 ± 3%, *n* = 9; *p* = 0.0002, one-way ANOVA with Bonferroni *post hoc* test), further confirming a slowdown in slow endocytosis.

Second, we investigated whether activation of TrkB receptors was involved in BDNF-induced presynaptic inhibition. We applied ANA-12 (500 nm), a potent and selective TrkB receptor antagonist (Montalbano et al., 2013), with BDNF (100 ng/ml) in the extracellular solution. After incubating for 30 min, the BDNF-induced inhibition of calcium influx, exocytosis, and endocytosis after depol_20ms_ was abolished, confirming that the effect of BDNF was mediated by the activation of TrkB receptors (Fig. 2*D*). Incubation with only ANA-12 did not affect the presynaptic ICa and exocytosis/endocytosis (Fig. 2*D*).

Next, we examined whether BDNF-TrkB signaling can retrogradely activate presynaptic CB1Rs to inhibit the presynaptic ICa and exocytosis/endocytosis. We applied 1 μm of AM251, the CB1R antagonist (Lemtiri-Chlieh and Levine, 2010), in the extracellular solution in the presence of BDNF (100 ng/ml). We found that, after incubation for 30 min, BDNF modulation of the ICa and exocytosis/endocytosis was no longer apparent after depol_20ms_ (ICa: 2.1 ± 0.1 nA, *p* > 0.9999; ΔCm: 509 ± 27 fF, *p* > 0.9999; Rate_endo_: 45 ± 3 fF/s, *p* > 0.9999; ΔCm_15s_%: 34 ± 3%, *p* > 0.9999; one-way ANOVA with Bonferroni *post hoc* test for all four groups; *n* = 7; Fig. 2*C*, blue, *D*), suggesting that CB1R was the downstream effector in BDNF-inhibited synaptic transmission. Furthermore, adding only AM251 to the extracellular solution did not result in any difference from control (Fig. 2*D*).

To confirm the activation of CB1Rs in BDNF-induced presynaptic inhibition, we included 2 μm WIN55212-2, the exogenous cannabinoid agonist (Lemtiri-Chlieh and Levine, 2010), in the extracellular solution to investigate whether it can mimic the inhibitory effect of BDNF. After incubation for 30 min, we found that both ICa and vesicle exocytosis/endocytosis were dramatically inhibited to a level similar to BDNF-treated calyces after depol_20ms_ (ICa: 1.4 ± 0.1 nA, *p* > 0.9999; ΔCm: 367 ± 22 fF, *p* > 0.9999; Rate_endo_: 25 ± 2 fF/s, *p* > 0.9999; ΔCm_15s_%: 44 ± 2%, *p* > 0.9999; one-way ANOVA with Bonferroni *post hoc* test for all four groups; *n* = 8; Fig. 2*C*, green, *D*). An additional 1 μm AM251 fully abolished the WIN-induced inhibition of ICa and vesicle exocytosis/endocytosis (Fig. 2*D*). These results confirm that BDNF inhibits synaptic transmission via activation of presynaptic CB1Rs.

We further investigated the involvement of presynaptic and/or postsynaptic TrkB receptors by specifically blocking the postsynaptic TrkB receptors. We applied K252a (200 nm), another TrkB receptor inhibitor, to the postsynaptic pipette solution in a whole-cell configuration, and then recorded the ICa and vesicle exocytosis/endocytosis at the presynaptic nerve terminal of the same synapse after bath application of BDNF (100 ng/ml) for 20 min. Inhibition of postsynaptic TrkB receptors in the presence of 100 ng/ml BDNF abolished the depol_20ms_-induced inhibition of ICa and ΔCm (ICa: 2.1 ± 0.1 nA, *p* > 0.9999; ΔCm: 503 ± 36 fF, *p* > 0.9999; one-way ANOVA with Bonferroni *post hoc* test), and subsequent endocytosis recovered (Rate_endo_: 49 ±4 fF/s, *p* > 0.9999; ΔCm_15s_%: 29 ± 4%, *p* > 0.9999; one-way ANOVA with Bonferroni *post hoc* test; *n* = 7; Fig. 2*E*,*F*, blue). Administration of K252a in the postsynaptic pipette solution did not affect the presynaptic ICa and vesicle exocytosis/endocytosis (Fig. 2*E*,*F*, black). Therefore, we concluded that BDNF induces presynaptic inhibition via activation of postsynaptic TrkB receptors at calyces.

### BDNF inhibits rapid endocytosis via the eCB signaling pathway

After exocytosis, the fused presynaptic membrane is retrieved via endocytosis to maintain efficient synaptic transmission and normal morphology of the presynaptic nerve terminal (L. G. Wu et al., 2014). Two forms of endocytosis are commonly observed at calyces: clathrin- and dynamin-dependent slow endocytosis, and clathrin-independent dynamin-dependent rapid endocytosis (W. Wu et al., 2005; X. S. Wu et al., 2009). We examined whether BDNF also affects rapid endocytosis.

**Figure 3.**
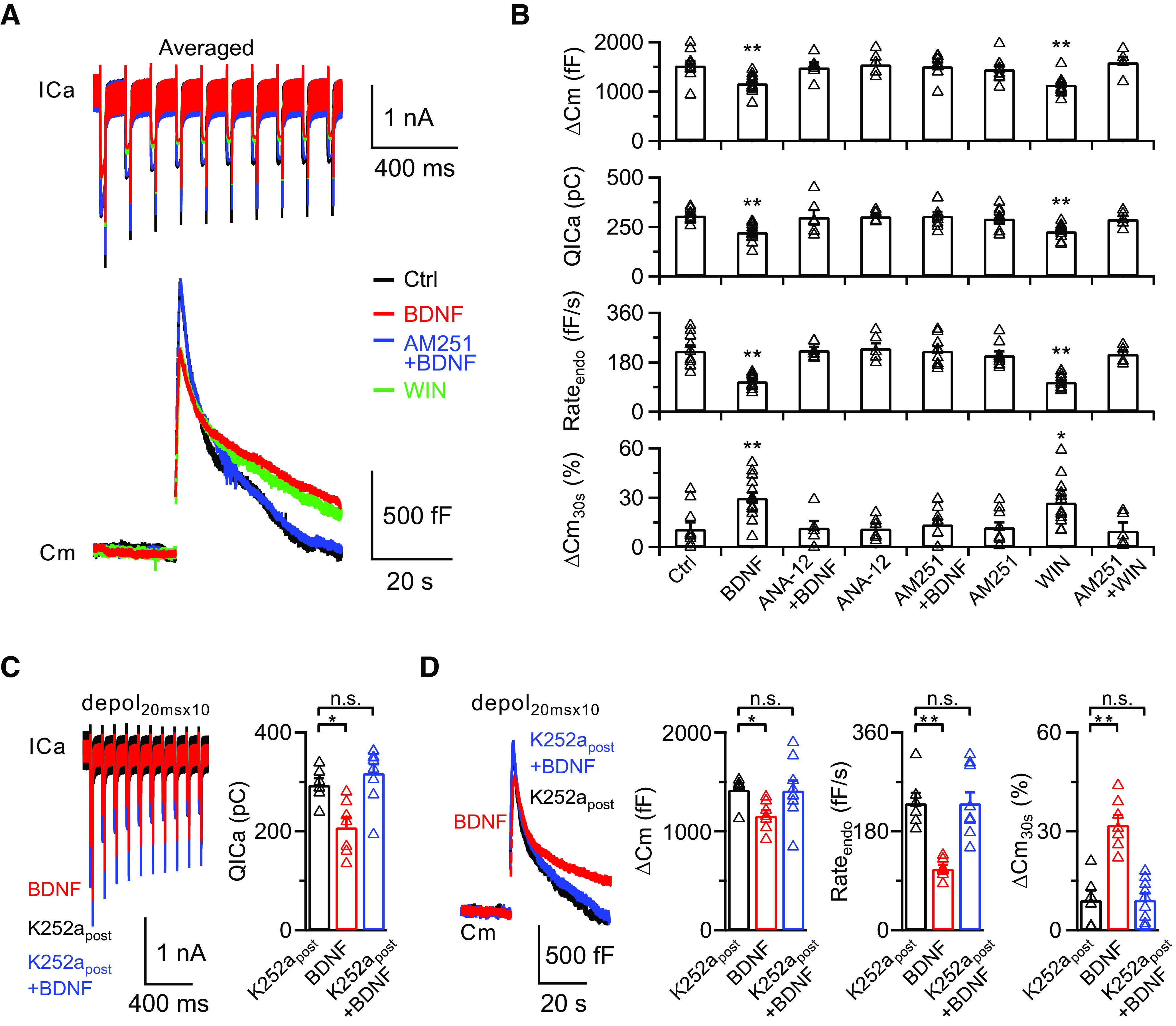
BDNF inhibits rapid endocytosis via the eCB signaling pathway. ***A***, Averaged presynaptic ICa (top) and Cm (bottom) induced by depol_20msx10_ in the control (Ctrl, black), BDNF treatment (BDNF, red), BDNF treatment in the presence of AM251 (AM251+BDNF, blue), and WIN55212-2 treatment (WIN, green) groups. ***B***, Statistics for ΔCm, QICa, Rate_endo_, and ΔCm_30s_% from different treatments in extracellular solution (Ctrl, *n* = 10; BDNF, *n* = 13; ANA-12+BDNF, *n* = 6; ANA-12, *n* = 6; AM251+BDNF, *n* = 9; AM251, *n* = 9; WIN, *n* = 12; AM251+WIN, *n* = 5). *p* values were calculated using a one-way ANOVA with Bonferroni's multiple comparisons test. *p < 0.05. **p < 0.01. ***C***, Left, Averaged presynaptic ICa induced by depol_20msx10_ in the control (K252a_post_, 200 nm K252a in the postsynaptic pipette solution, *n* = 6; black), BDNF treatment (BDNF, without K252a in the postsynaptic pipette solution in the presence of BDNF, *n* = 7; red), and BDNF treatment with K252a (K252a_post_+BDNF, 200 nm K252a in the postsynaptic pipette solution in the presence of BDNF, *n* = 9; blue) groups. Right, Statistics for QICa in all three groups using a one-way ANOVA with Bonferroni's multiple comparisons test. *p < 0.05. ***D***, Left, Averaged Cm induced by depol_20msx10_ from ***C***. Right, Statistics for ΔCm, Rate_endo_, and ΔCm_30s_% in all three groups using a one-way ANOVA with Bonferroni's multiple comparisons test. *p < 0.05. **p < 0.01. n.s., not significant. Detailed statistical information is provided in Table 1.

We applied a stronger stimulation of 10 depol_20ms_ at 10 Hz (depol_20msx10_) to induce rapid endocytosis at calyces. In control p8-p10 rats, depol_20msx10_ induced a much larger calcium influx (QICa; 308 ± 10 pC) and a higher ΔCm (1527 ± 94 fF, *n* = 10; Fig. 3*A*, top, black), representing more vesicle release. The capacitance decayed biexponentially with a rapid and slow τ of 1.9 ± 0.1 s and 16.4 ± 1.6 s, respectively (*n* = 10; Fig. 3*A*, bottom, black). In the presence of BDNF (100 ng/ml) in the extracellular solution, depol_20msx10_ evoked a reduced calcium influx (224 ± 12 pC, *p* = 0.0002; one-way ANOVA with Bonferroni *post hoc* test) and smaller ΔCm (1166 ± 49 fF, *p* = 0.0021; one-way ANOVA with Bonferroni *post hoc* test; *n* = 13; Fig. 3*A*, red). Both rapid and slow endocytosis on depol_20msx10_ was greatly inhibited (control: Rate_endo_, 222 ± 19 fF/s; ΔCm_30s_%, 11 ± 4%; *n* = 10; BDNF: Rate_endo_, 111 ± 7 fF/s, *p* < 0.0001; ΔCm_30s_%, 30 ± 4%, *p* = 0.0025; one-way ANOVA with Bonferroni *post hoc* test; *n* = 13; Fig. 3*A*, bottom), confirming that BDNF can also inhibit clathrin-independent rapid endocytosis.

Similarly, when we applied ANA-12 (500 nm) with BDNF (100 ng/ml) in the extracellular solution for 30 min, the calcium influx, exocytosis, and endocytosis evoked by depol_20msx10_ were not obviously different from those in the control group (Fig. 3*B*), showing that the BDNF-induced inhibition of rapid endocytosis was mediated by activation of TrkB receptors. We further examined the involvement of CB1Rs in the BDNF-induced inhibition of rapid endocytosis. After incubation with AM251 (1 μm) in the presence of BDNF (100 ng/ml) in the extracellular solution for 30 min, the calcium influx, exocytosis, and rapid/slow endocytosis recovered after depol_20msx10_ (QICa: 307 ± 20 pC, *p* > 0.9999; ΔCm: 1513 ± 74 fF, *p* > 0.9999; Rate_endo_: 221 ± 17 fF/s, *p* > 0.9999; ΔCm_30s_%: 14 ± 3%, *p* > 0.9999; one-way ANOVA with Bonferroni *post hoc* test for all four groups; *n* = 9; Fig. 3*A*, blue). In addition, 2 μm WIN55212-2 in the extracellular solution for 30 min mimicked the effect of BDNF after depol_20msx10_ (QICa: 228 ± 11 pC, *p* > 0.9999; ΔCm: 1142 ± 52 fF, *p* > 0.9999; Rate_endo_: 109 ± 6 fF/s, *p* > 0.9999; ΔCm_30s_%: 27 ± 4%, *p* > 0.9999; one-way ANOVA with Bonferroni *post hoc* test for all four groups; *n* = 12; Fig. 3*A*, green), which can be fully abolished by the addition of 1 μm AM251 (Fig. 3*B*). These results clearly demonstrate that BDNF inhibits clathrin-independent rapid endocytosis via activation of the presynaptic CB1Rs. Similarly, we confirmed that the inhibition of rapid endocytosis was mediated by activation of postsynaptic TrkB receptors. We obtained paired recordings during specific inhibition of the postsynaptic TrkB receptors by applying K252a (200 nm) in the postsynaptic pipette solution and found that the depol_20msx10_-induced inhibition of calcium influx and exocytosis/endocytosis in the presence of BDNF (100 ng/ml) was abolished (QICa: 318 ± 18 pC, *p* > 0.9999; ΔCm: 1425 ± 93 fF, *p* > 0.9999; Rate_endo_: 231 ± 20 fF/s, *p* > 0.9999; ΔCm_30s_%: 9 ± 2%, *p* > 0.9999; one-way ANOVA with Bonferroni *post hoc* test for all four groups; *n* = 9; Fig. 3*C*,*D*).

**Figure 4.**
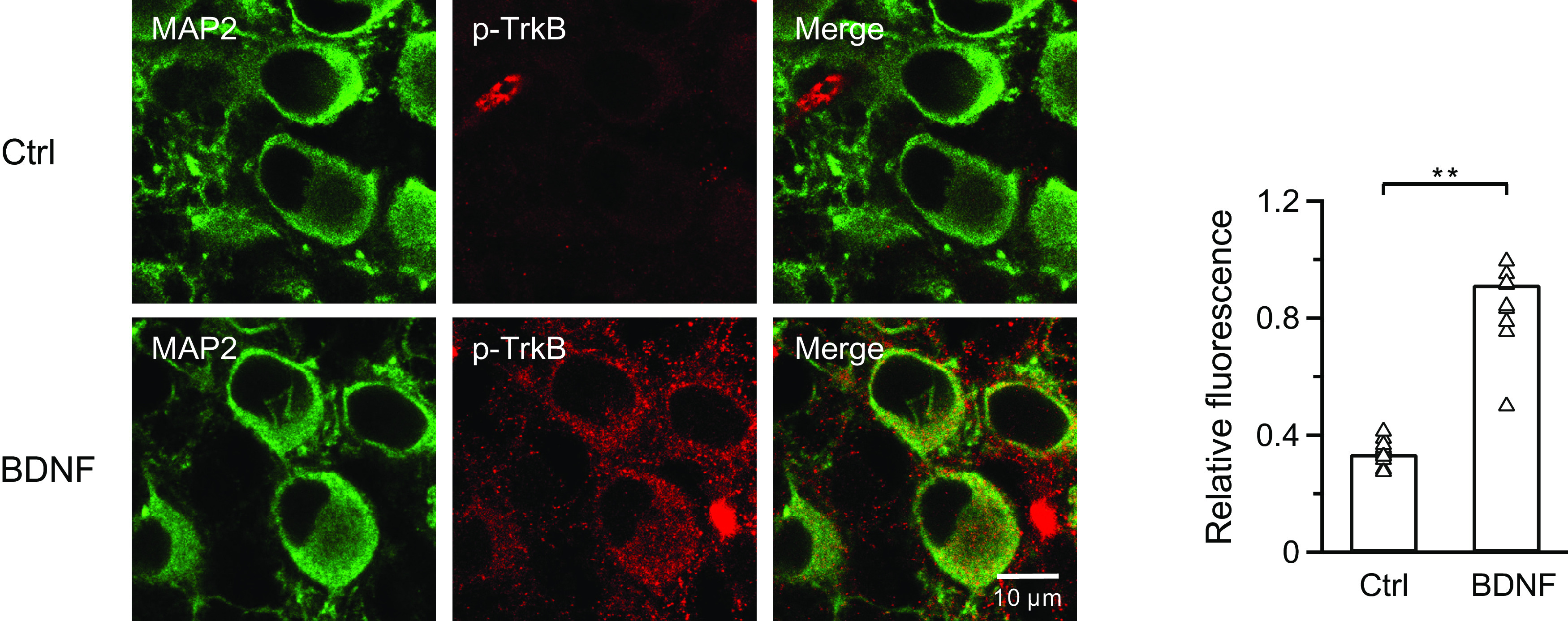
BDNF activates postsynaptic TrkB receptors. Left, Immunostaining of postsynaptic neurons (MAP2, green) and phospho-TrkB receptors (red) at calyces. Scale bar, 10 μm. Right, Statistics for relative fluorescence before (*n* = 15 cells from 3 experiments) and after (*n* = 11 cells from 3 experiments) application of BDNF (100 ng/ml) using an unpaired Student's *t* test. **p < 0.01.

The presynaptic inhibition of ICa and vesicle exocytosis/endocytosis demonstrated above critically depend on the activation of postsynaptic TrkB receptors. Therefore, we verified TrkB activation in MNTB principal neurons using immunohistochemistry. We chose MAP2 as the marker for postsynaptic neurons and SC-8058 as the phospho-TrkB marker. Minimal phospho-TrkB receptor was observed in the control group (except some staining of blood vessels; Fig. 4, left). In the BDNF-treated group, the phospho-TrkB receptors dramatically increased at the postsynaptic neurons (Ctrl: 34 ± 1%, *n* = 15; BDNF: 92 ± 7%, *n* = 11; *p* < 0.0001, unpaired *t* test; Fig. 4), confirming the activation of postsynaptic TrkB receptors.

### Postsynaptic release of eCBs is required for the BDNF-induced presynaptic inhibition

**Figure 5.**
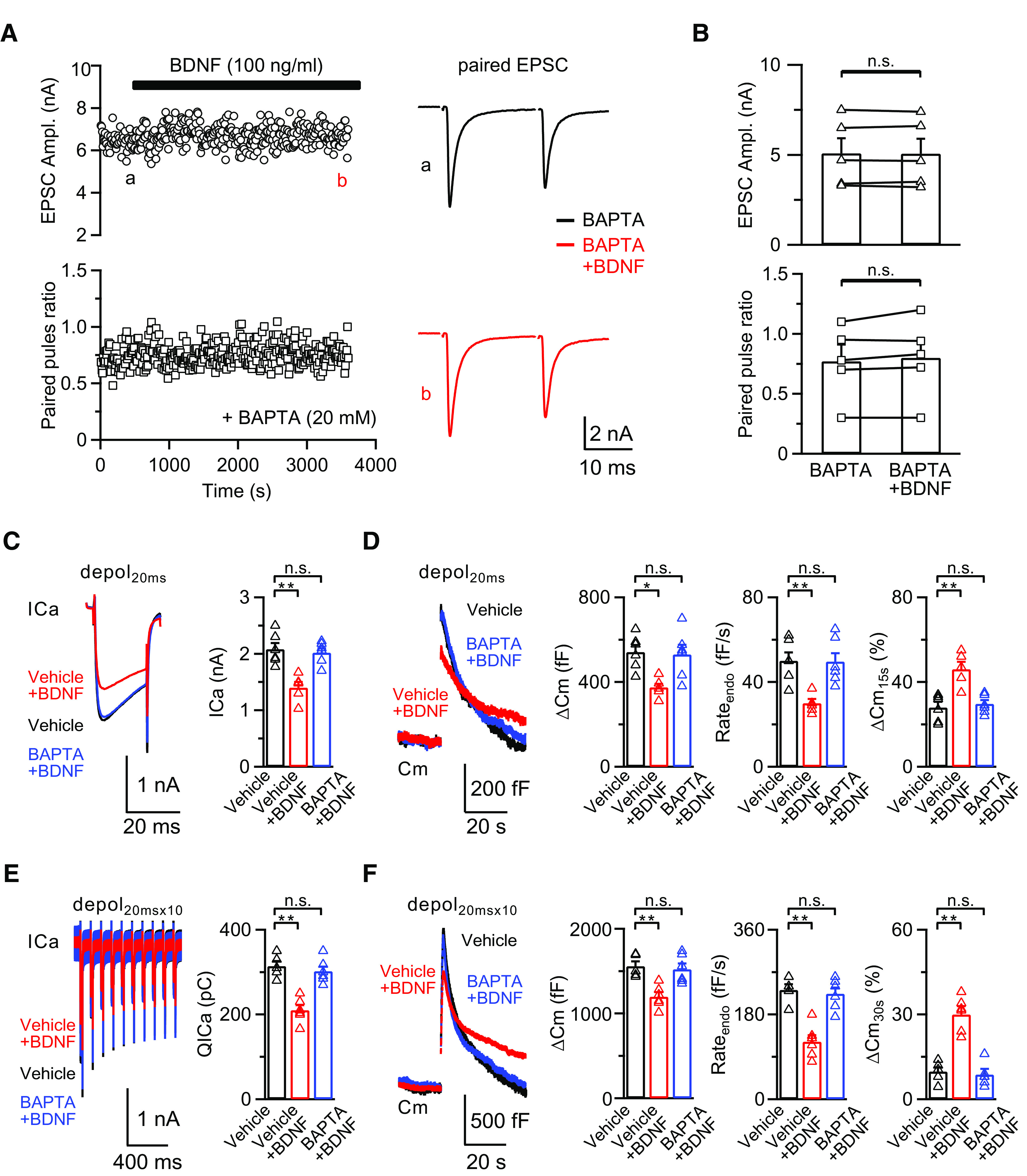
The BDNF-induced inhibitory effect is postsynaptic calcium-dependent. ***A***, Left, Sampled paired EPSC recordings and corresponding PPR in response to 0.1 Hz fiber stimulation at the midline of the trapezoid body; 20 mm BAPTA was included in the postsynaptic pipette solution to chelate free calcium ions; 100 ng/ml BDNF was added to the extracellular solution after obtaining a stable baseline and EPSCs were recorded for another 30-60 min. Right, Sampled EPSCs at time points a (baseline, black) and b (BDNF treatment, red) from left. ***B***, Statistics for EPSC amplitude and PPR (*n* = 5) from ***A*** using a paired Student's *t* test. ***C***, Left, Averaged presynaptic ICa induced by depol_20ms_ in the control (Vehicle, without BAPTA in the postsynaptic pipette solution, *n* = 6; black), BDNF treatment (Vehicle+BDNF, without BAPTA in the postsynaptic pipette solution in the presence of BDNF, *n* = 5; red), and BDNF treatment with BAPTA (BAPTA+BDNF, 20 mm BAPTA in the postsynaptic pipette solution in the presence of BDNF, *n* = 6; blue) groups. All recordings were made in the paired-recording mode. Right, Statistics for ICa in all three groups using a one-way ANOVA with Bonferroni's multiple comparisons test. **p < 0.01. ***D***, Left, Averaged Cm induced by depol_20ms_ from ***C***. Right, Statistics for ΔCm, Rate_endo_, and ΔCm_15s_% in all three groups using a one-way ANOVA with Bonferroni's multiple comparisons test. *p < 0.05. **p < 0.01. ***E***, ***F***, Similar to ***C***, ***D***, except the stimulation was depol_20msx10_ (Vehicle, *n* = 5; Vehicle+BDNF, *n* = 6; BAPTA+BDNF, *n* = 6). **p < 0.01. n.s., not significant. Detailed statistical information is provided in Table 1.

Having shown that BDNF acts on the postsynaptic TrkB receptors and retrogradely induces presynaptic inhibition of the ICa and exocytosis/endocytosis via activation of the presynaptic CB1Rs, we investigated the involvement of postsynaptic eCB synthesis. The eCBs have been shown to be released from postsynaptic neurons via Ca^2+^-dependent mechanisms (Kushmerick et al., 2004). We recorded the EPSCs with 20 mm BAPTA in the postsynaptic pipette solution to examine whether the BDNF-induced inhibition of synaptic transmission could be abolished. After 30-60 min bath application of BDNF (100 ng/ml), the EPSC amplitude was not changed (baseline: 5.1 ± 0.8 nA, BDNF: 5.1 ± 0.8 nA; *p* = 0.8688, paired *t* test; *n* = 5; Fig. 5*A*,*B*), confirming that the release of eCBs is required for BDNF-induced inhibitory effect.

To examine the presynaptic effect when calcium is chelated, we included 0 (vehicle) or 20 mm BAPTA in the postsynaptic pipette solution via a whole-cell configuration, and then applied BDNF (100 ng/ml) in the extracellular solution. After 20 min, we performed presynaptic recordings at the nerve terminal of the same synapse to examine the calcium influx and exocytosis/endocytosis. We found that 20 mm BAPTA can completely abolish the BDNF-induced inhibition of presynaptic calcium influx and exocytosis/endocytosis after either depol_20ms_ (ICa: 2.0 ± 0.1 nA, *p* > 0.9999; ΔCm: 530 ± 46 fF, *p* > 0.9999; Rate_endo_: 50 ± 4 fF/s, *p* > 0.9999; ΔCm_15s_%: 30 ± 2%, *p* > 0.9999; one-way ANOVA with Bonferroni *post hoc* test for all four groups; *n* = 6) or depol_20msx10_ (QICa: 302 ± 12 pC, *p* > 0.9999; ΔCm: 1522 ± 69 fF, *p* > 0.9999; Rate_endo_: 224 ± 13 fF/s, *p* > 0.9999; ΔCm_30s_%: 9 ± 2%, *p* > 0.9999; one-way ANOVA with Bonferroni *post hoc* test for all four groups; *n* = 6; Fig. 5*C–F*).

**Figure 6.**
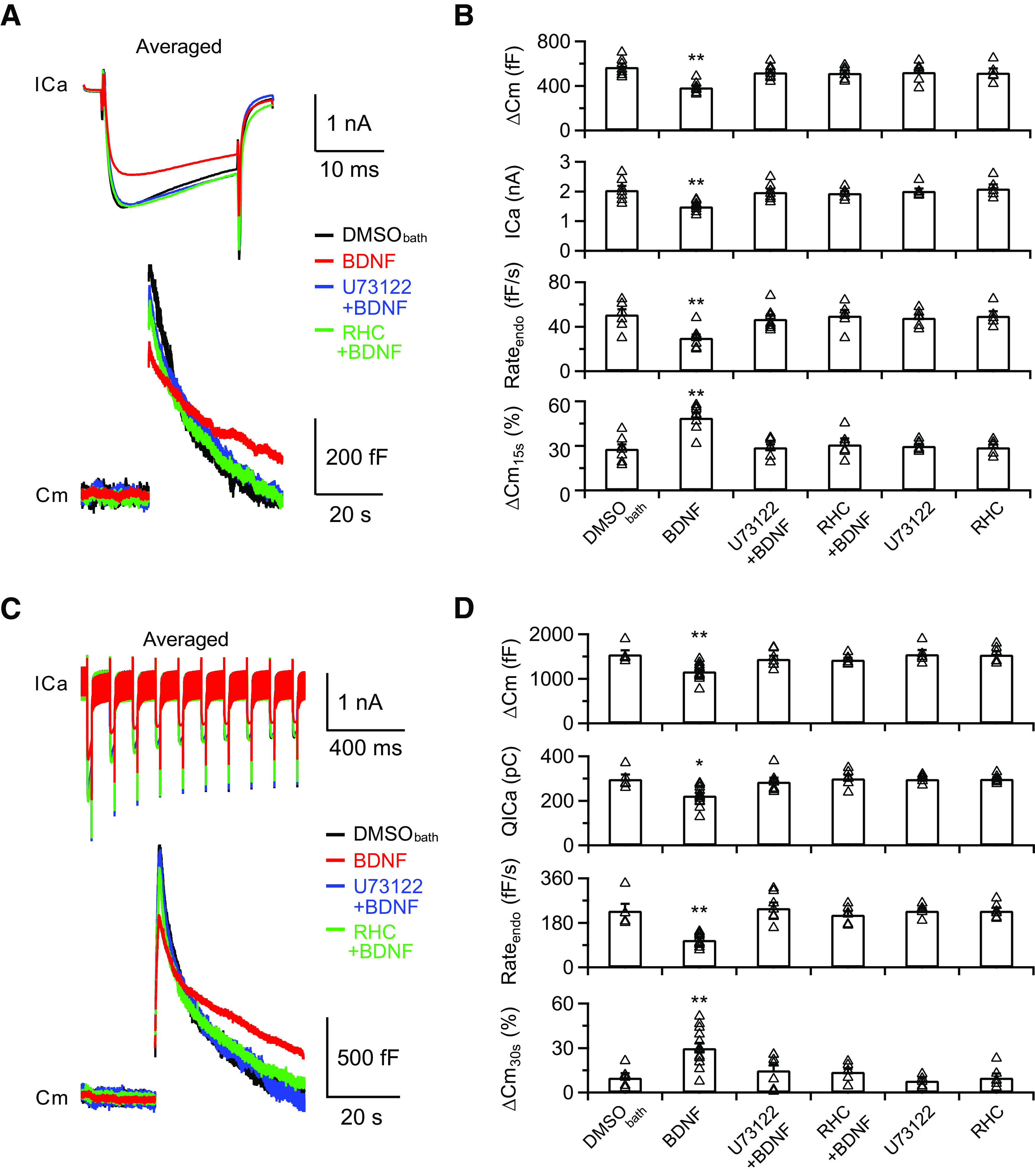
Postsynaptic release of eCBs is required for the inhibitory effects of BDNF. ***A***, Averaged presynaptic ICa (top) and Cm (bottom) induced by depol_20ms_ in the control (DMSO_bath_, 0.1% DMSO in the extracellular solution, black), BDNF treatment (BDNF, identical to Fig. 2*C*), BDNF treatment in the presence of U73122 (U73122+BDNF, blue), and BDNF treatment in the presence of RHC80267 (RHC+BDNF, green) groups. ***B***, Statistics for ΔCm, ICa, Rate_endo_, and ΔCm_15s_% with different treatments in extracellular solution (DMSO_bath_, *n* = 7; BDNF, identical to Fig. 2*C*, *n* = 8; U73122+BDNF, *n* = 7; RHC+BDNF, *n* = 6; U73122, *n* = 6; RHC, *n* = 5). The *p* values were calculated using a one-way ANOVA with Bonferroni's multiple comparisons test. **p < 0.01. ***C***, ***D***, Similar to ***A***, ***B***, except the stimulation was depol_20msx10_ (DMSO_bath_, *n* = 5; BDNF, identical to Fig. 3*A*, *n* = 13; U73122+BDNF, *n* = 7; RHC+BDNF, *n* = 6; U73122, *n* = 5; RHC, *n* = 6). *p < 0.05. **p < 0.01. Detailed statistical information is provided in Table 1.

Next, we disrupted the synthesis of 2-arachidonoylcglycerol, the principal eCB for activity-dependent retrograde signaling, by blocking phospholipase C (PLC) or diacylglycerol lipase (DGL), both of which are key postsynaptic enzymes in generating 2-arachidonoylcglycerol (Ohno-Shosaku et al., 2012; Anderson et al., 2015). After application of U73122 (5 μm), an inhibitor of PLC (Rinaldo and Hansel, 2013), with BDNF (100 ng/ml) for 30 min, we found that the BDNF-inhibited ICa and exocytosis/endocytosis induced by depol_20ms_ were fully abolished (ICa: 2.0 ± 0.1 nA, *p* > 0.9999; ΔCm: 522 ± 24 fF, *p* > 0.9999; Rate_endo_: 47 ± 4 fF/s, *p* > 0.9999; ΔCm_15s_%: 29 ± 2%, *p* > 0.9999; one-way ANOVA with Bonferroni *post hoc* test for all four groups; *n* = 7; Fig. 6*A*, blue, *B*). When we induced rapid endocytosis with depol_20msx10_, the calcium influx, exocytosis, and subsequent rapid endocytosis were also similar to control (QICa: 286 ± 18 pC, *p* > 0.9999; ΔCm: 1447 ± 73 fF, *p* > 0.9999; Rate_endo_: 243 ± 22 fF/s, *p* > 0.9999; ΔCm_30s_%: 16 ± 5%, *p* > 0.9999; one-way ANOVA with Bonferroni *post hoc* test for all four groups; *n* = 7; Fig. 6*C*, blue, *D*). We performed similar experiments with application of 30 μm RHC80267, a DGL inhibitor (Lemtiri-Chlieh and Levine, 2010), in the presence of BDNF (100 ng/ml) and obtained similar results as control (Fig. 6*A*,*C*, green; Table 1). Application of only U73122 or RHC80267 did not induce any significant change from control (Fig. 6*B*,*D*). These results further confirm that postsynaptic eCB synthesis is required for BDNF-induced presynaptic inhibition.

### BDNF inhibits endocytosis in calcium-dependent and -independent ways

**Figure 7.**
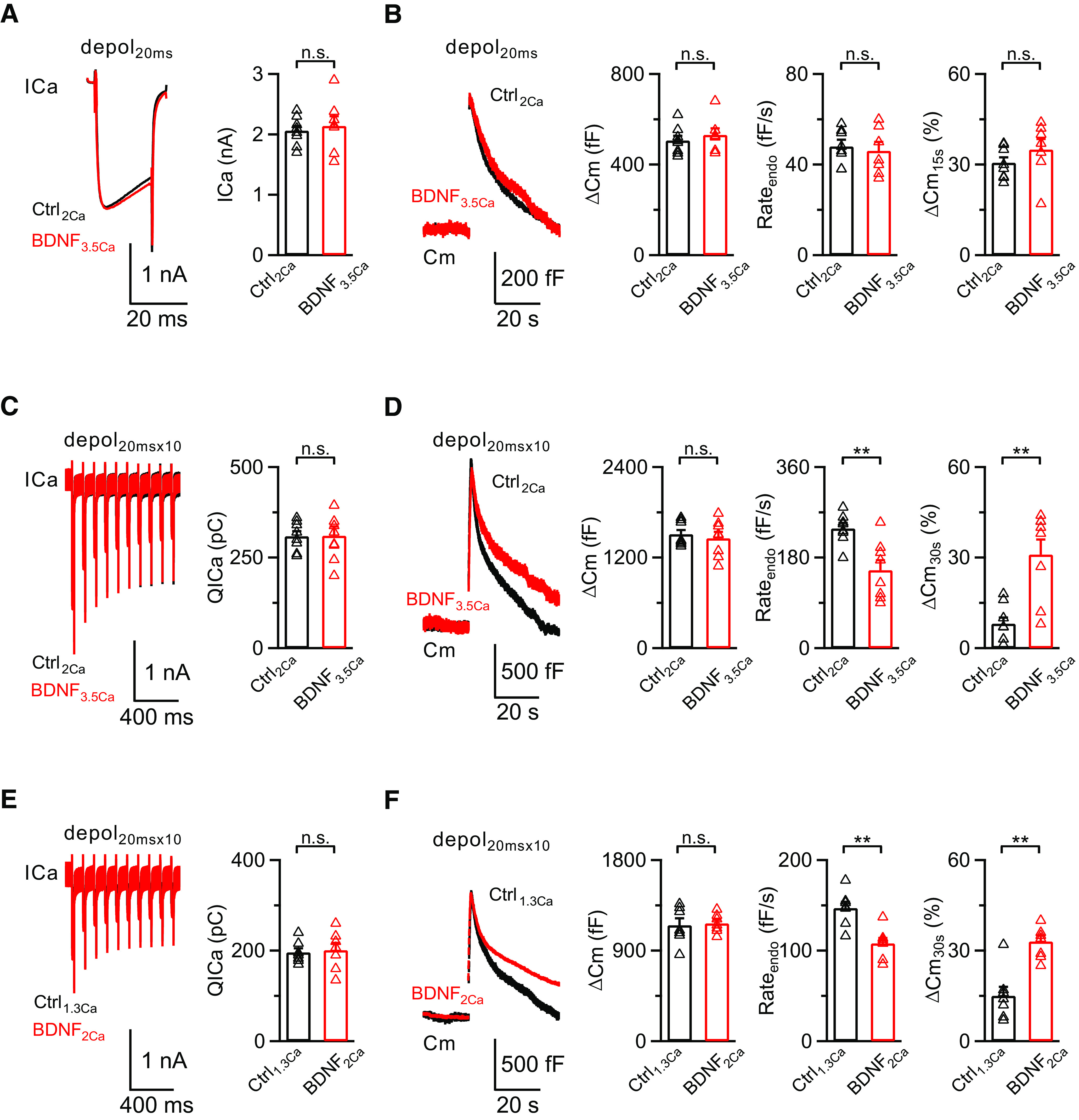
BDNF inhibits endocytosis in a calcium-dependent and -independent manner. ***A***, Left, Averaged presynaptic ICa induced by depol_20ms_ in the control group (Ctrl_2Ca_, *n* = 8; black) and BDNF treatment with 3.5 mm extracellular calcium (BDNF_3.5Ca_, *n* = 7; red). Right, Statistics for ICa in two groups using an unpaired Student's *t* test. ***B***, Left, Averaged Cm induced by depol_20ms_ from ***A***. Right, Statistics for ΔCm, Rate_endo_, and ΔCm_15s_% in two groups using an unpaired Student's *t* test. ***C***, ***D***, Similar to ***A***, ***B***, except the stimulation was depol_20msx10_ (Ctrl_2Ca_, *n* = 8; BDNF_3.5Ca_, *n* = 8). **p < 0.01. ***E***, Left, Averaged presynaptic ICa induced by depol_20msx10_ in the control group with 1.3 mm extracellular calcium (Ctrl_1.3Ca_, *n* = 7; black) and BDNF treatment with 2 mm extracellular calcium (BDNF_2Ca_, *n* = 7; red). Right, Statistics for QICa in two groups using an unpaired Student's *t* test. ***F***, Left, Averaged Cm induced by depol_20msx10_ from ***E***. Right, Statistics for ΔCm, Rate_endo_, and ΔCm_30s_% in two groups using an unpaired Student's *t* test. **p < 0.01. n.s., not significant. Detailed statistical information is provided in Table 1.

As BDNF can inhibit presynaptic calcium influx and subsequent exocytosis/endocytosis, which is consistent with our previous finding that calcium triggers exocytosis and initiates all forms of endocytosis (X. S. Wu et al., 2009; Xue et al., 2012b), we further investigated whether BDNF can directly modulate presynaptic vesicle endocytosis in a calcium-independent manner. To address this issue, we increased the extracellular calcium concentration to counterbalance the BDNF-induced reduction in the ICa. In the presence of BDNF (100 ng/ml) in the extracellular solution with 3.5 mm calcium, the ICa and exocytosis induced by depol_20ms_ were similar to control (Ctrl_2Ca_: ICa, 2.1 ± 0.1 nA; ΔCm, 506 ± 21 fF; *n* = 8; BDNF_3.5Ca_: ICa, 2.2 ± 0.2 nA, *p* = 0.6351; ΔCm, 531 ± 29 fF, *p* = 0.4757; unpaired *t* test; *n* = 7; Fig. 7*A*,*B*). The Rate_endo_ and ΔCm_15s_% were slightly reduced, but still not significantly different from control (Ctrl_2Ca_: Rate_endo_, 48 ± 3 fF/s; ΔCm_15s_%, 31 ± 2%; *n* = 8; BDNF_3.5Ca_: Rate_endo_, 46 ± 4 fF/s, *p* = 0.6434; ΔCm_15s_%, 35 ± 4%, *p* = 0.2413; unpaired *t* test; *n* = 7; Fig. 7*B*). However, endocytosis was still inhibited (Ctrl_2Ca_: Rate_endo_, 238 ± 11 fF/s; ΔCm_30s_%, 8 ± 2%; *n* = 8; BDNF_3.5Ca_: Rate_endo_, 155 ± 20 fF/s, *p* = 0.0029; ΔCm_30s_%, 31 ± 5%, *p* = 0.0008; unpaired *t* test; *n* = 8; Fig. 7*D*) when we applied depol_20msx10_ to induce rapid endocytosis, although calcium influx and exocytosis were similar to control in the presence of BDNF (100 ng/ml) with 3.5 mm extracellular calcium (Ctrl_2Ca_: QICa, 309 ± 14 pC; ΔCm, 1510 ± 57 fF; *n* = 8; BDNF_3.5Ca_: QICa, 310 ± 26 pC, *p* = 0.7844; ΔCm, 1457 ± 85 fF, *p* = 0.6111; unpaired *t* test; *n* = 8; Fig. 7*C*,*D*). These results confirm that, in addition to calcium-dependent regulation of slow and rapid endocytosis, BDNF can also directly modulate the rapid form of endocytosis in a calcium-independent manner. We also performed similar experiments with 1.3 mm calcium in the extracellular solution of the control group to mimic the BDNF-reduced calcium influx. Although calcium influx and exocytosis were reduced to a level similar to the BDNF-treated group, endocytosis was still significantly inhibited in the presence of BDNF (100 ng/ml) after depol_20msx10_ (Ctrl_1.3Ca_: Rate_endo_, 147 ± 7 fF/s; ΔCm_30s_%, 15 ± 3%; *n* = 7; BDNF_2Ca_: Rate_endo_, 108 ± 6 fF/s, *p* =0.0018; ΔCm_30s_%, 33 ± 2%, *p* = 0.0004; unpaired *t* test; *n* = 7; Fig. 7*E*,*F*). Therefore, we concluded that BDNF can modulate presynaptic vesicle endocytosis in both a calcium-dependent and -independent manner.

### The AC/PKA signaling pathway is involved in CB1R-induced presynaptic inhibition

Many studies have suggested that activation of CB1R modulates synaptic transmission by reducing neurotransmitter release at both excitatory and inhibitory synapses (Heifets and Castillo, 2009; Kano et al., 2009; Ohno-Shosaku et al., 2012). However, how CB1R activation leads to the inhibition of synaptic transmission remains elusive (de Jong and Verhage, 2009). A previous study demonstrated that the AC/PKA signaling pathway modulates endocytosis in response to strong stimulation (Yao and Sakaba, 2012). Therefore, we investigated whether AC/PKA are also involved in BDNF-TrkB-CB1R-induced presynaptic inhibition.

**Figure 8.**
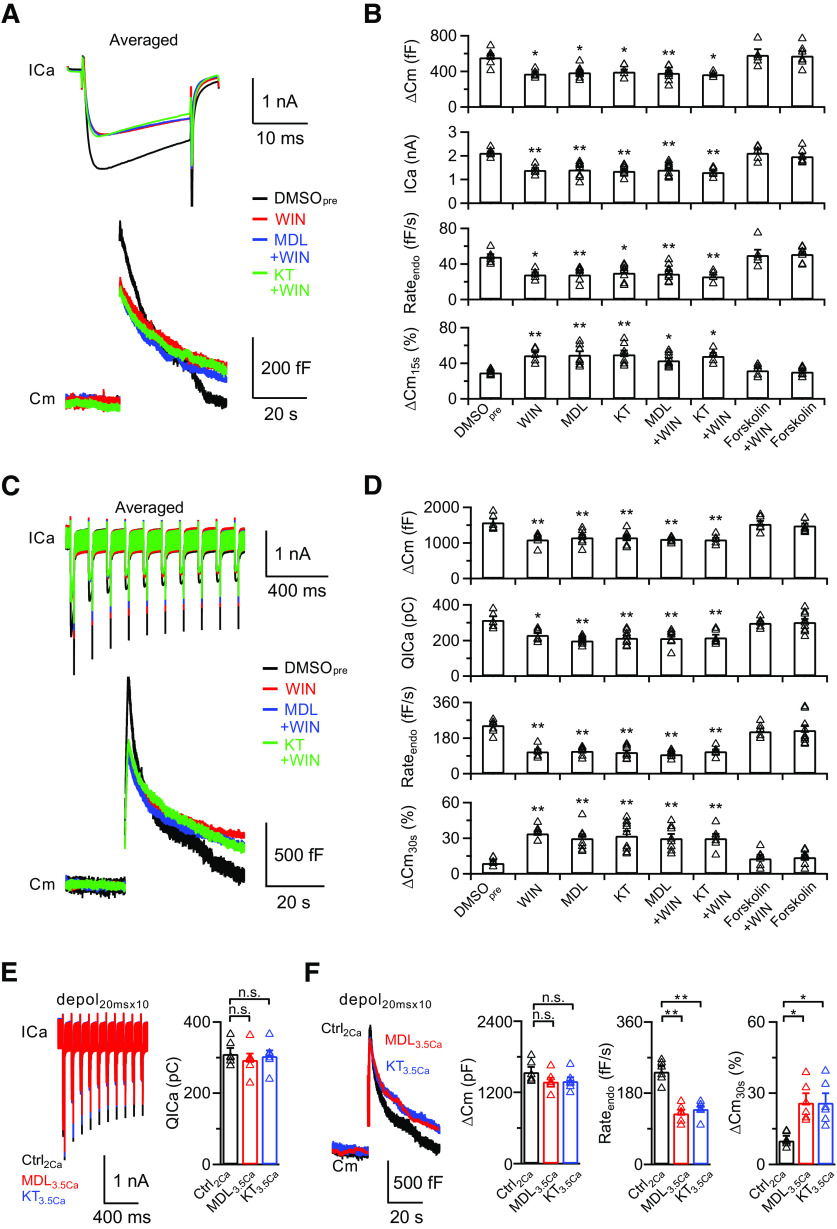
The AC/PKA signaling pathway is involved in the inhibitory effects of BDNF. ***A***, Averaged presynaptic ICa (top) and Cm (bottom) induced by depol_20ms_ in the control (DMSO_pre_, 0.1% DMSO in the presynaptic pipette solution, black), WIN55212-2 treatment (WIN, red), WIN55212-2 treatment in the presence of MDL 12330A (MDL+WIN, blue), and WIN55212-2 treatment in the presence of KT 5720 (KT+WIN, green) groups. ***B***, Statistics for ΔCm, ICa, Rate_endo_, and ΔCm_15s_% from different treatments in extracellular solution (DMSO_pre_, *n* = 6; WIN, *n* = 5; MDL, *n* = 8; KT, *n* = 7; MDL+WIN, *n* = 9; KT+WIN, *n* = 5; Forskolin+WIN, *n* = 5; Forskolin, *n* = 7). *p* values were calculated using a one-way ANOVA with Bonferroni's multiple comparisons test. *p < 0.05. **p < 0.01. ***C***, ***D***, Similar to ***A***, ***B***, except the stimulation was depol_20msx10_ (DMSO_pre_, *n* = 6; WIN, *n* = 6; MDL, *n* = 8; KT, *n* = 9; MDL+WIN, *n* = 8; KT+WIN, *n* = 6; Forskolin+WIN, *n* = 6; Forskolin, *n* = 9). *p < 0.05. **p < 0.01. ***E***, Left, Averaged ICa induced by depol_20msx10_ in the control (Ctrl_2Ca_, identical to DMSO_pre_ in Fig. 8*C*, *n* = 6; black), MDL 12330A treatment with 3.5 mm extracellular calcium (MDL_3.5Ca_, *n* = 6; red), and KT 5720 treatment with 3.5 mm extracellular calcium (KT_3.5Ca_, *n* = 6; blue) groups. Right, Statistics for QICa using a one-way ANOVA with Bonferroni's multiple comparisons test. ***F***, Left, Averaged Cm induced by depol_20msx10_ from ***E***. Right, Statistics for ΔCm, Rate_endo_, and ΔCm_30s_% using a one-way ANOVA with Bonferroni's multiple comparisons test. *p < 0.05. **p < 0.01. n.s., not significant. Detailed statistical information is provided in Table 1.

We applied specific AC and PKA antagonists MDL (10 μm) and KT5720 (2 μm), respectively (Yao and Sakaba, 2012), to the presynaptic pipette solution. Presynaptic calcium influx, exocytosis, and endocytosis induced by depol_20ms_ or depol_20msx10_ were dramatically suppressed (Fig. 8*A–D*; Table 1). When we applied the exogenous cannabinoid agonist WIN55212-2 (2 μm) with MDL or KT5720, we did not detect further inhibition of calcium influx and exocytosis/endocytosis (Fig. 8*A–D*; Table 1), suggesting that AC/PKA signaling was involved in the BDNF-TrkB-CB1R-induced presynaptic inhibition at calyces. We also examined the effect of forskolin, a potent adenylate cyclase activator (Ho et al., 2015; Rey et al., 2020). In the presence of 50 μm forskolin in the presynaptic pipette solution, depol_20ms_ or depol_20msx10_ induced similar calcium influx and exocytosis/endocytosis to the control (DMSO_pre_; Fig. 8*A*,*C*). An additional 2 μm WIN55212-2 in the presence of forskolin did not induce any inhibitory effect (Fig. 8*B*,*D*; Table 1), confirming the involvement of the AC/PKA pathway. Interestingly, when we increased the extracellular calcium concentration to 3.5 mm, application of MDL (10 μm) or KT5720 (2 μm) still partially inhibited the endocytosis rate after depol_20msx10_ (MDL_3.5Ca_: Rate_endo_, 129 ± 8 fF/s, *p* = 0.0002; ΔCm_30s_%, 25 ± 4%, *p* = 0.0332; *n* = 6; KT_3.5Ca_: Rate_endo_, 139 ± 8 fF/s, *p* = 0.0011; ΔCm_30s_%, 26 ± 5%, *p* = 0.0456; *n* = 6; one-way ANOVA with Bonferroni *post hoc* test; Fig. 8*E*,*F*), which is consistent with calcium-dependent and -independent BDNF-induced modulation (Fig. 7). Therefore, our results confirmed the involvement of the AC/PKA signaling pathway in the BDNF-TrkB-eCB-induced inhibition of synaptic transmission.

**Figure 9.**
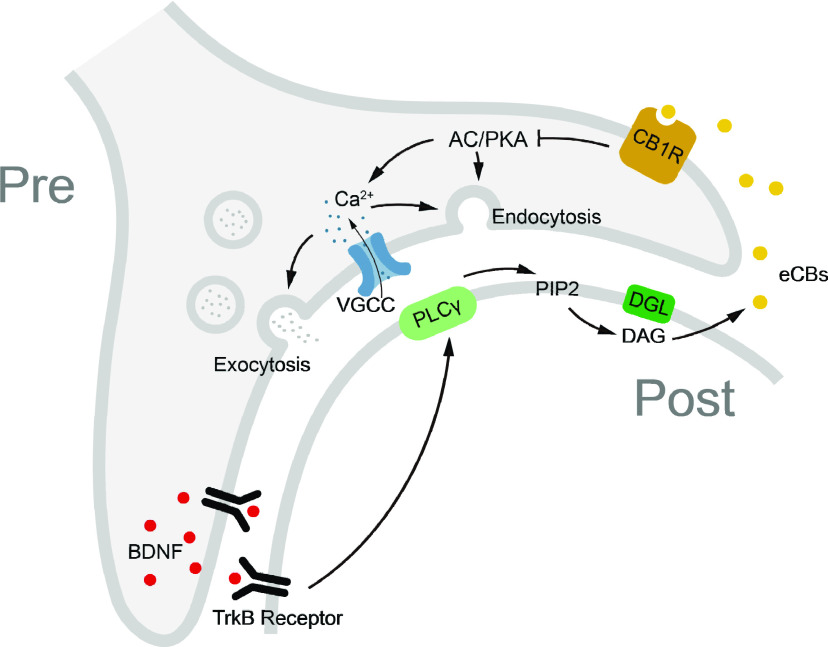
Schematic of the proposed signaling pathway for BDNF-inhibited synaptic transmission. BDNF activates postsynaptic TrkB receptors to induce eCB release via the PLCγ/DGL pathway. eCBs retrogradely bind to presynaptic CB1Rs and lead to suppression of the AC/PKA signaling pathway, finally inhibiting presynaptic calcium influx and exocytosis/endocytosis. VGCC, Voltage-gated calcium channel; PIP_2_, phosphatidylinositol 4,5-bisphosphate; DAG, diacylglycerol.

## Discussion

BDNF and eCBs are widely expressed neuromodulators that play crucial roles in various neuronal functions, plasticity, and physiological processes. In the present study, we found that BDNF inhibits presynaptic calcium influx and vesicle exocytosis/endocytosis via the BDNF-TrkB-CB1R signaling pathway. BDNF selectively activates postsynaptic TrkB receptors to evoke the release of eCBs via the PLCγ/DGL pathway that retrogradely activates presynaptic CB1Rs, leading to the suppression of downstream AC/PKA signaling. Our study suggests a new mechanism of BDNF-induced inhibition of synaptic transmission at the calyx synapse (Fig. 9).

### BDNF induces a retrograde eCB signaling pathway

How does activation of postsynaptic TrkB receptors lead to inhibition of presynaptic calcium influx and exocytosis/endocytosis? In the present study, we report the involvement of the eCB signaling pathway in BDNF-induced inhibition of synaptic transmission at calyces. Inhibition of the presynaptic CB1R or disruption of postsynaptic eCB synthesis abolishes the BDNF-induced presynaptic inhibition, and exogenous cannabinoid agonist WIN55212-2 can mimic the inhibitory effect of BDNF, demonstrating BDNF-induced retrograde presynaptic inhibition.

AC and PKA are widely reported to be involved in G-protein-activated presynaptic inhibition (Chevaleyre et al., 2007; Castillo et al., 2012). Here, we report that inhibition of AC/PKA leads to suppression of calcium influx and vesicle exocytosis/endocytosis at calyces. Activation of AC by 50 μm forskolin in the bath solution fully abolished the WIN-induced presynaptic inhibition (Fig. 8*A–D*). Previous studies have reported that activation of AC by forskolin potentiates synaptic transmission in many different preparations by different mechanisms (Kaneko and Takahashi, 2004; Cheung et al., 2006; Yao and Sakaba, 2010; Renner et al., 2017). However, the forskolin-induced potentiation of EPSC has been shown to occur independent of calcium and RRP size (Ariel et al., 2012). A detailed study at calyx synapses also demonstrated that increased cAMP concentration leads to a large increase in release probability and much smaller increases in RRP size (Yao and Sakaba, 2010), which is consistent with forskolin not affecting the calcium influx and vesicle exocytosis/endocytosis. Together, our results indicate that the BDNF-induced reduction in calcium influx and vesicle exocytosis/endocytosis is mediated by the retrograde eCB signaling pathway.

### BDNF inhibits endocytosis via calcium-dependent and -independent pathways

The previous study reported that BDNF inhibits slow and rapid endocytosis via a calcium-independent pathway because BDNF does not reduce the QICa on mild or intense stimulation (Baydyuk et al., 2015). However, in our study, we found that BDNF inhibits calcium influx and exocytosis/endocytosis on either depol_20ms_ or depol_20msx10_ (Figs. 2*C*,*D*, 3*A*,*B*). When we increased the extracellular calcium concentration in the BDNF-treated group or decreased the calcium concentration in the control group to induce similar amounts of calcium influx and vesicle exocytosis in the two groups, endocytosis was still partially inhibited because of direct modulation of endocytosis by the AC/PKA pathway. Therefore, our findings suggest that BDNF inhibits presynaptic endocytosis in both calcium-dependent and -independent ways.

### Physiologic implication of the BDNF-TrkB-eCB signaling cascade

Many studies have shown that BDNF can facilitate the efficacy of excitatory synapses by altering either presynaptic neurotransmitter release (Carmignoto et al., 1997; Jovanovic et al., 2000) or the magnitude of postsynaptic responses (Alder et al., 2005) in brain slices or cultured neurons. However, several studies have shown that BDNF may play a different role in the brainstem (Balkowiec et al., 2000; Clark et al., 2011). For example, in the brainstem nucleus tractus solitarius slice, BDNF can reduce the amplitude of mEPSC, the evoked EPSC, and the action potential discharge, indicating reduced intrinsic neuronal excitability (Clark et al., 2011).

BDNF/eCB-induced inhibition of neurotransmission may serve as negative feedback to provide activity-dependent neuroprotection from excitotoxicity. Depolarization-induced suppression, a strong depolarization of postsynaptic neurons leading to reduced synaptic transmission via the release of eCBs, has been interpreted as an efficient means of neuronal protection (Wilson and Nicoll, 2001). Excessive glutamate release has also been shown to promote the synthesis of eCBs to avoid hyperexcitability (Kushmerick et al., 2004).

BDNF/eCB signaling may also exert neuroprotective effects on neurodegenerative diseases, such as Huntington's disease. Delivery and overexpression of BDNF or activation of CB1R protect the striatal neurons from excitotoxicity, reduce motor disorders, and prevent the loss of medium spiny neurons (Kells et al., 2008; Blázquez et al., 2011; Connor et al., 2016; Aymerich et al., 2018).

A recent study reported that a reduction in BDNF expression impairs synaptic transmission at the calyx of Held (Jang et al., 2019). However, an increase in BDNF may modulate synaptic transmission via the activation of different signaling cascades, including the inhibition of synaptic transmission via eCB signaling shown here and in other studies (Lemtiri-Chlieh and Levine, 2010; Zhao and Levine, 2014; Zhong et al., 2015). Furthermore, increased BDNF expression has been observed in many physiological or pathologic conditions. For example, a protective mechanism of the CB1R-dependent increase in BDNF expression has been reported in mice with kainate-induced seizures (Marsicano et al., 2003). At calyces, the basal neuronal firing can be increased to >600–800 Hz on stimulation (Von Gersdorff and Borst, 2002; Hermann et al., 2007), which may increase the BDNF level in an activity-dependent manner (Y. J. Wu et al., 2004; Singer et al., 2014) to induce inhibitory neuroprotection from excitotoxicity. A recent study showed that, in the lower part of the auditory system in the brain, BDNF may improve the signal-to-noise ratio and sound sensitivity by increasing the inhibitory strength of neurons at hearing onset. A significant increase in central noise can be observed after auditory nerve injury (Chumak et al., 2016).

In conclusion, we examined the presynaptic mechanisms and signaling cascades of BDNF-induced inhibition of synaptic transmission at a glutamatergic central synapse. By uncovering the detailed mechanisms underlying how BDNF/TrkB couples with the eCB signaling pathway to modulate synaptic transmission, our study may provide a comprehensive understanding of how BDNF and eCBs associate in an overlapping set of neurologic diseases.
